# Development and internal validation of an algorithm for estimating mortality in patients encountered by physician-staffed helicopter emergency medical services

**DOI:** 10.1186/s13049-024-01208-y

**Published:** 2024-04-23

**Authors:** Emil Reitala, Mitja Lääperi, Markus B. Skrifvars, Tom Silfvast, Hanna Vihonen, Pamela Toivonen, Miretta Tommila, Lasse Raatiniemi, Jouni Nurmi

**Affiliations:** 1 Department of Anaesthesia, Intensive Care and Pain Medicine, University of Helsinki and Helsinki University Hospital, PO Box 340, FI-00029 Helsinki, HUS Finland; 2 Department of Emergency Medicine and Services, University of Helsinki and Helsinki University Hospital, PO Box 340, FI-00029 Helsinki, HUS Finland; 3 Emergency Medical Services, Centre for Prehospital Emergency Care, Department of Emergency, Anaesthesia and Pain Medicine, Tampere University Hospital, PO Box 2000, FI-33521 Tampere, Finland; 4 Department of Emergency Medicine and Services, Päijät-Häme Central Hospital, FI-15850 Lahti, Finland; 5 Centre for Prehospital Care, Institute of Clinical Medicine, Kuopio University Hospital, PO Box 100, FI-70029 Kuopio, KYS Finland; 6 Department of Perioperative Services, Intensive Care Medicine and Pain Management, Turku University Hospital and University of Turku, PO Box 52, FI-20521 Turku, Finland; 7 HEMS unit, Division for prehospital emergency care, Oulu University Hospital, Lentokentäntie 670, FI-09460 Oulunsalo, Finland; 8 Research Group of Surgery, Anaesthesiology and Intensive Care, Division of Anaesthesiology, Oulu University Hospital, Medical Research Centre, University of Oulu, PO Box FI-90029, Oulu, Finland

**Keywords:** Risk prediction model, Air ambulances, Critical care, Mortality, Emergency medical services, Pre-hospital, Benchmarking

## Abstract

**Background:**

Severity of illness scoring systems are used in intensive care units to enable the calculation of adjusted outcomes for audit and benchmarking purposes. Similar tools are lacking for pre-hospital emergency medicine. Therefore, using a national helicopter emergency medical services database, we developed and internally validated a mortality prediction algorithm.

**Methods:**

We conducted a multicentre retrospective observational register-based cohort study based on the patients treated by five physician-staffed Finnish helicopter emergency medical service units between 2012 and 2019. Only patients aged 16 and over treated by physician-staffed units were included. We analysed the relationship between 30-day mortality and physiological, patient-related and circumstantial variables. The data were imputed using multiple imputations employing chained equations. We used multivariate logistic regression to estimate the variable effects and performed derivation of multiple multivariable models with different combinations of variables. The models were combined into an algorithm to allow a risk estimation tool that accounts for missing variables. Internal validation was assessed by calculating the optimism of each performance estimate using the von Hippel method with four imputed sets.

**Results:**

After exclusions, 30 186 patients were included in the analysis. 8611 (29%) patients died within the first 30 days after the incident. Eleven predictor variables (systolic blood pressure, heart rate, oxygen saturation, Glasgow Coma Scale, sex, age, emergency medical services vehicle type [helicopter vs ground unit], whether the mission was located in a medical facility or nursing home, cardiac rhythm [asystole, pulseless electrical activity, ventricular fibrillation, ventricular tachycardia vs others], time from emergency call to physician arrival and patient category) were included. Adjusted for optimism after internal validation, the algorithm had an area under the receiver operating characteristic curve of 0.921 (95% CI 0.918 to 0.924), Brier score of 0.097, calibration intercept of 0.000 (95% CI -0.040 to 0.040) and slope of 1.000 (95% CI 0.977 to 1.023).

**Conclusions:**

Based on 11 demographic, mission-specific, and physiologic variables, we developed and internally validated a novel severity of illness algorithm for use with patients encountered by physician-staffed helicopter emergency medical services, which may help in future quality improvement.

**Supplementary Information:**

The online version contains supplementary material available at 10.1186/s13049-024-01208-y.

## Background

Since the release of the Acute Physiology and Chronic Health Evaluation (APACHE) score in 1981 [1], several prognostic scoring systems have been developed to assess the severity of disease in critically ill patients treated in the intensive care unit (ICU) [2, 3]. Risk scores have also been developed for other purposes, such as the assessment of the severity of injury or a given disease, facilitation of triage decisions and to indicate the need for interventions [2]. ICU risk scores may be used to detect and quantify organ failure and to provide a statistical estimation of outcomes for quality improvement, audit and benchmarking purposes [3–5]. The APACHE score, Simplified Acute Physiology Score (SAPS) and Mortality Prediction Model (MPM) are examples of the latter [1, 3, 6, 7].

Care of critically ill patients is often initiated in pre-hospital settings, and in certain patient populations this care is paramount for patient outcomes [8–13]. Even so, the risk stratification tools used in the pre-hospital setting are mostly limited to disease-specific risk scores and early warning scores (EWS) used for triage decision making, identifying critical illness and assessing the level-of-care requirements for the receiving centre [14–17]. Different EWS have shown varying values in predicting short-term adverse outcomes in pre-hospital settings, with decreasing predictive abilities during longer follow-up [13, 15, 17, 18]. We currently lack a uniform mortality risk model for the wide range of critically ill pre-hospital patients attended by physician-staffed units that could allow for the estimation of standardised mortality ratios (SMR) in benchmarking and for risk stratification in epidemiological studies. Using a national helicopter emergency medical services (HEMS) database, we developed and internally validated a uniform risk algorithm for predicting mortality in patients treated by physician-staffed HEMS (P-HEMS) units based on essential physiological variables and additional factors independent of treatment.

## Methods

### Study setting

To develop a mortality model, we conducted a multicentre retrospective observational register-based cohort study of patients encountered by the national Finnish helicopter emergency medical services (FinnHEMS) between January 2012 and September 2019. The FinnHEMS organisation is publicly funded and comprises six operational units, of which five are physician-staffed and one is staffed only by paramedics. The physician-staffed units operate within the catchment areas of the five Finnish university hospitals (see Additional file 1). The paramedic-staffed unit operates solely in the sparsely populated district of Lapland. The service areas cover most of the population of Finland [19]. The fleet includes Airbus 135 and 145 helicopters, as well as rapid response ground vehicles that are used in short-range missions and whenever the weather conditions are not suitable for aviation.

Finnish P-HEMS units mainly encounter critically ill or injured patients who require pre-hospital critical care. The P-HEMS units are dispatched based on uniform predefined criteria by the national emergency response centre agency. Ambulance crews can also request P-HEMS response. The major categories for P-HEMS dispatch include major trauma, cardiac arrest, and impaired level of consciousness. The physician can cancel or deny the mission if the patient is not considered able to benefit from the care provided by the P-HEMS based on the information provided by the dispatcher or ambulance crew. The HEMS physicians are mostly experienced anaesthesiologists. Secondary transfers are rare, but the units can be dispatched to medical facilities or nursing homes for primary missions. The characteristics of the Finnish HEMS, including the relatively low utilization of helicopter transportation of the patients, have been recently described [20].

We report our findings in accordance with the Transparent Reporting of a multivariable prediction model for Individual Prognosis Or Diagnosis (TRIPOD) statement [21]. Ethical approval was not required for this study, as it was retrospective in nature, exclusively utilizing non-original register-based data that were neither generated nor collected specifically for this research and involved no interventions or direct contact with study participants.

The philosophical underpinnings of this research are based on addressing the current gaps in risk assessment tools for pre-hospital critical care. This research paradigm stresses the importance of evidence-based medicine and using predictive analytics to improve pre-hospital care delivery. The theoretical framework builds upon established risk scoring systems used in critical care settings and expands their application to the pre-hospital environment. By adhering to transparent reporting standards, we aim to ensure the robustness and applicability of the developed risk algorithm.

### Participants and study outcome

**Fig. 1:**
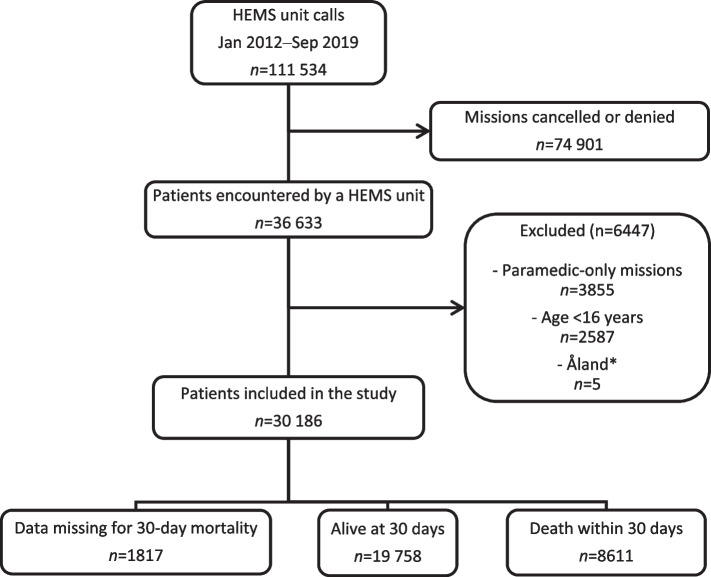
Study cohort selection process. HEMS, helicopter emergency medical services. *Missions including patients located in the autonomous region of Åland

We included patients aged 16 years and over who were assessed by P-HEMS units. Patients treated by the paramedic-staffed unit operating in Lapland were not included due to differences in staffing and dispatch criteria [20]. Patients from the autonomous region of Åland were excluded as the local health care system functions in separation from the mainland. No other eligibility criteria were applied (Fig. 1). Our main outcome was overall mortality within 30 days of encountering the P-HEMS unit. This was chosen in preference to mortality during shorter follow-up as we consider long-term survival to be an outcome of greater importance for both the individual and the society.

### Data collection

The research material was derived from the FinnHEMS database (FHDB), covering every HEMS mission in Finland during the study period. Since its adoption for nationwide use in 2012, the operational and clinical data from every FinnHEMS mission have been registered and stored in the FHDB in accordance with generally accepted guidelines and registry templates for pre-hospital data collection [20, 22–24]. The FHDB contains records with a total of more than 170 variables (see Additional file 2). The data are manually entered into the database by a member of the FinnHEMS unit that attended the mission. Input errors with obvious abnormal measures are disallowed by the FHDB registration system; however, errors within the normal range of each measure are not detected. For physiological parameters, only the first measurements after the HEMS arrival were included.

Population registry data provided by the Finnish Digital and Population Data Services Agency were used to obtain information about the main outcome and verify the age and sex of the patient. A personal identity code offered by the Digital and Population Data Services Agency links a nationwide population registry with healthcare software systems.

### Candidate predictor variables

From the FHDB, 14 candidate variables were selected for analysis based on the consensus of the authors: patient age, number of patients in a single mission, patient sex, Glasgow Coma Scale (GCS), HEMS vehicle type (helicopter or ground unit), cardiac rhythm, respiratory rate, systolic blood pressure, oxygen saturation, heart rate, patient category, time from emergency call to HEMS arrival, time from emergency call to arrival of the first emergency medical services (EMS) unit and whether the mission was located in a medical facility or nursing home. The consensus was reached by employing a collaborative decision-making approach among the authors, who independently selected potential variables from a comprehensive list of variables. Subsequently, the selections were compiled, and similarities and differences were assessed collectively. Through iterative discussion and consensus-building, agreement was reached.

### Missing data

We dealt with missing data by using multiple imputations with chained equations [25]. This method estimates missing data over multiple iterations to create complete datasets for analysis. We performed 10 iterations to generate 30 complete datasets. The differences between the patients with and without missing data were used to identify further variables to be included in the imputation, along with the candidate predictor variables and the primary outcome. In two cases, clearly erroneous data were observed, which were treated as missing data.

### Model development and predictor effects

We analysed how patient characteristics relate to the outcome using Mann-Whitney U test. Then, we used multivariate logistic regression to estimate the effects of the studied predictors on the outcome in each of the 30 imputed datasets. To combine these results from various datasets, we used Rubin's rule, a commonly used formula to combine results from multiply imputed data [25]. With this method, we obtained the final pooled estimate for the effects of the predictors.

To avoid the excessive influence of extreme values, we applied winsorization method to all continuous variables (except for the GCS) adjusting the values by 1% at both ends. Any values below or above these limits were set to the limit itself. Additionally, we used a technique called restricted cubic splines to examine how continuous predictor variables interact with the outcome in a non-linear way. We used three knots for GCS and four for the remaining continuous variables. To assess the statistical significance of individual variable effects on the outcome, we utilised the Wald test.

### Algorithm formation

We aimed to develop a tool that could be used with actual pre-hospital data, in which missing values are frequent. To allow the use with incomplete data, we did not only create a prediction model but also multiple additional models with different combinations of the same predictor variables that we used in the original model. These predictor combinations were designed so that each additional model excluded one or more of the candidate predictor variables with the most missing data.

All these models were then combined into an algorithm, the Critical HEMS Algorithm for Mortality Prediction (CHAMP). The algorithm allows for the case-by-case selection of a tailored model for each individual based on the available variables. The CHAMP algorithm automatically selects the model with the most available variables for each patient. All models were built in the same manner as the original model with no missing variables (referred to as the full model later in the text).

### Assessing the performance

The discriminative abilities of both the models and the algorithm were investigated using the area under the receiver operating characteristic (AUROC) curve. Calibration was evaluated by fitting a calibration curve and calculating the slopes and intercepts for the predicted probability of the outcome. The slope of one and the intercept of zero would suggest ideal calibration. Overall performances were assessed using the Brier score, a metric used to measure the accuracy of predictions, encompassing both discrimination and calibration. It ranges from zero to one, with zero indicating perfect accuracy. In addition, The Hosmer–Lemeshow test was used to test the calibration of the algorithm, as nonsignificant values imply a good fit.

For individual models, all performance estimates were calculated in the imputed sets and pooled using Rubin’s rules, whereas the performance of the CHAMP was calculated for the original population to illustrate a more authentic user experience. We used a generalised additive model (GAM) risk plot, a receiver operating characteristic plot and a risk decile plot to visualise the performance and calibration of the algorithm.

### Sensitivity analysis and internal validation

As the studied population included a notable degree of cardiac arrest patients, a specific subgroup known to have high mortality [26], we performed a planned sensitivity analysis excluding patients with cardiac arrest as the primary dispatch code to assess the robustness of the results.

Internal validation was performed by calculating the optimism of each performance estimate using the von Hippel method with four imputed sets each containing 250 bootstrapped samples.

### Software

Analyses were performed with R version 4.1.0 [27] using the *mice* [25], *rms* [28] and *bootImpute*packages [29]. The plots were constructed using the *ggplot2* [30] and *plotROC*packages [31].

## Results

### Study population

During the study period, 36 633 patients were encountered by the HEMS. After exclusions, 30 186 patients were included in the final analysis (Fig. 1). The median patient age was 60 [IQR 39 to 73] years, and 65% of the patients were male. The 30-day mortality rate was 30% (*n*=8611). A total of 11 971 (40%) of the patients included in the final analysis had missing data for at least one of the studied predictors or the outcome (see Additional file 3: Table S1). The study cohort is described in detail according to the occurrence of the main outcome in Table 1.

**Table 1:** Study population characteristics

	Alive at 30 days (*n*=19 758)	Death within 30 days (*n*=8611)	*p-*value	Missing data for 30-day mortality (*n*=1817)
Age, years	53.0 [33.4 to 68.4]	70.2 [60.0 to 79.8]	<0.001	50.0 [30.0 to 67.7]
Missing, n	0	0		0
Heart rate, beats per minute	90 [77 to 108]	90 [70 to 110]	<0.001	90 [77 to 105]
Missing, n	2529 (12.8)	5073 (58.9)		431 (23.7)
Systolic blood pressure, mmHg	131 [113 to 151]	134 [103 to 168]	0.008	130 [112 to 148]
Missing, n	2843 (14.4)	5273 (61.2)		489 (26.9)
Respiratory rate, breaths per minute	16 [14 to 20]	16 [12 to 22]	<0.001	16 [14 to 20]
Missing, n	6783 (34.3)	5880 (68.3)		867 (47.8)
Oxygen saturation, %	97 [94 to 99]	95 [89 to 98]	<0.001	97 [95 to 99]
Missing, n	3078 (15.6)	5506 (63.9)		482 (26.5)
Time to HEMS arrival, minutes	19 [14 to 29]	19 [14 to 28]	0.14	19 [13 to 28]
Missing, n	0	0		0
Time to EMS arrival, minutes	11 [8 to 16]	10 [7 to 15]	<0.001	11 [8 to 18]
Missing, n	13 427 (68.0)	4885 (56.7)		1330 (73.2)
GCS	14 [7 to 15]	3 [3 to 3]	<0.001	14 [7 to 15]
Missing, n	1284 (6.5)	763 (8.9)		151 (8.3)
Cardiac rhythm			<0.001	
Sinus rhythm	14 187 (71.8)	2287 (26.6)		1202 (66.2)
SVES, VES (mono)	85 (0.4)	30 (0.3)		6 (0.3)
AFlut, AFib, AV block (II–III), VES (poly)	1376 (7.0)	779 (9.0)		99 (5.4)
VF, VT, asystole, PEA	832 (4.2)	4683 (54.4)		79 (4.3)
Not registered	1620 (8.2)	132 (1.5)		198 (10.9)
Paced	94 (0.5)	79 (0.9)		5 (0.3)
Missing	1564 (7.9)	621 (7.2)		228 (12.5)
Medical facility or nursing home			<0.001	
No	18 727 (94.8)	7874 (91.4)		1757 (96.7)
Yes	1035 (5.2)	737 (8.6)		60 (3.3)
Missing	0	0		0
HEMS vehicle			0.004	
Ground unit^a^	9908 (50.1)	4158 (48.3)		929 (51.1)
Helicopter^b^	9850 (49.9)	4453 (51.7)		888 (48.9)
Missing	0	0		0
Number of patients			<0.001	
1	19 315 (97.8)	8577 (99.6)		1765 (97.1)
≥ 2	443 (2.2)	34 (0.4)		52 (2.9)
Missing	0	0		0
Sex			<0.001	
Female	7249 (36.7)	2791 (32.4)		531 (29.2)
Male	12 509 (63.3)	5750 (66.8)		1002 (55.1)
Missing	0	70 (0.8)		284 (15.6)
Patient category			<0.001	
Cardiac arrest	1404 (7.1)	5461 (63.4)		135 (7.4)
Trauma	6480 (32.8)	915 (10.6)		643 (35.4)
Respiratory failure	1084 (5.5)	294 (3.4)		75 (4.1)
Chest pain	810 (4.1)	75 (0.9)		48 (2.6)
Stroke	1125 (5.7)	821 (9.5)		82 (4.5)
Neurological^c^	2785 (14.1)	512 (5.9)		228 (12.5)
Psychiatric or intoxication	2941 (14.9)	63 (0.7)		308 (17.0)
Gynaecology and obstetrics	637 (3.2)	1 (0.0)		55 (3.0)
Infection	198 (1.0)	62 (0.7)		14 (0.8)
Other	2294 (11.6)	407 (4.7)		229 (12.6)
Missing	0	0		0

Data are median [IQR] or *n* (%). *AFib* Atrial fibrillation; *AFlut* Atrial flutter, *A*, Atrioventricular; *EMS* Emergency medical services, *GCS* Glasgow Coma Scale, *HEMS* Helicopter emergency medical services,*PEA* Pulseless electrical activity, *SVES* Supraventricular extrasystole, *VES* Ventricular extrasystole, *VF* Ventricular fibrillation, *VT* Ventricular tachycardia

^a^Rapid response vehicle + other

^b^HEMS helicopter + Border guard helicopter

^c^Other than stroke

### Model development

**Fig. 2:**
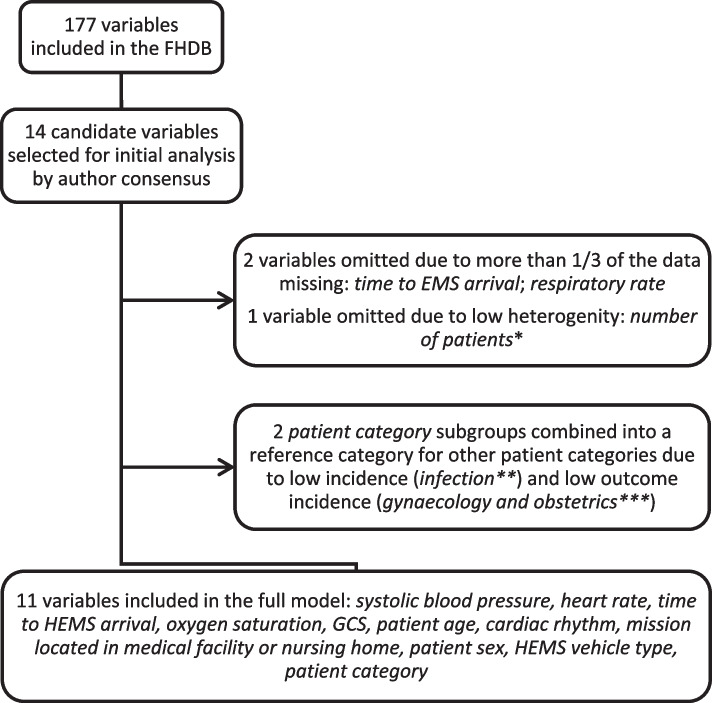
Selection of candidate predictors. GCS, Glasgow Coma Scale; EMS, emergency medical services; FHDB, FinnHEMS database; HEMS, helicopter emergency medical services. *≥ 2 patients encountered in 1.7% of the cases (cut-off limit 2.0%); **Patient category was *infection* in 260 (0.9%) cases (cut-off limit 2.0%); ***30-day mortality rate was 0.2% (*n*=1) in the patient category *gynaecology and obstetrics* (cut-off limit 2.0%)

**Fig. 3:**
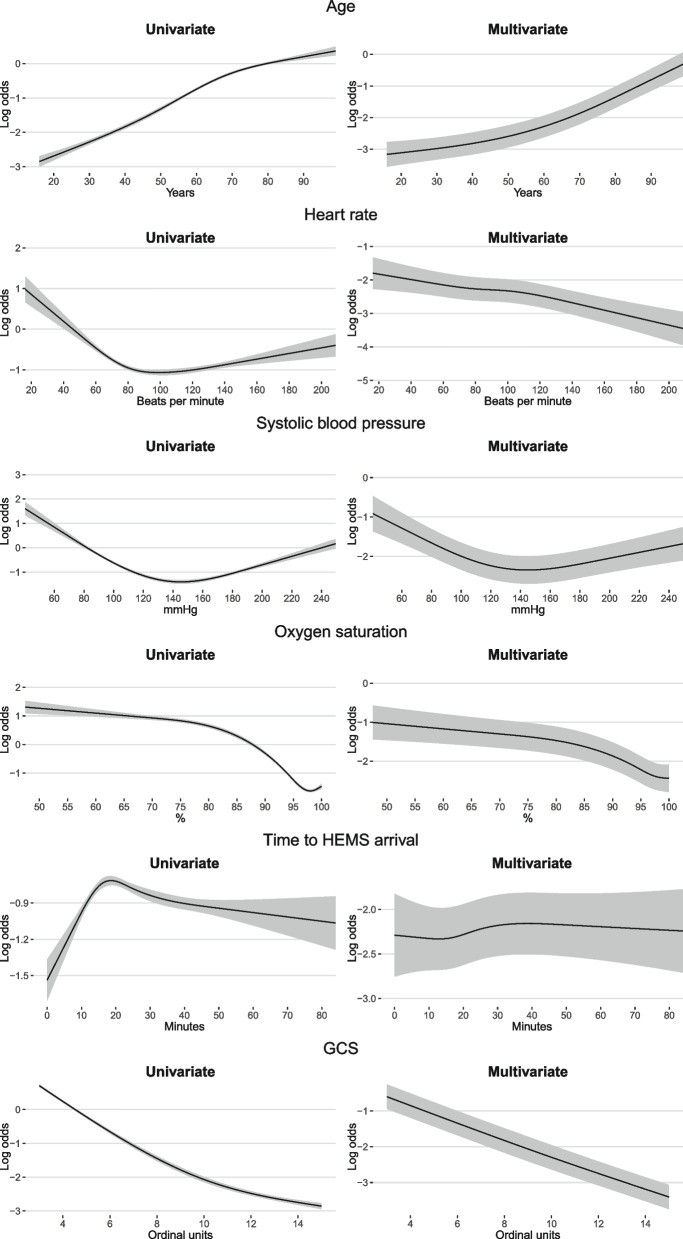
The association between 30-day mortality and selected continuous predictor variables. GCS, Glasgow Coma Scale; HEMS, helicopter emergency medical services

The variables were first screened for compatibility for the modelling and ones with too much missing data, too rare occurrences, or too few deaths per category were dropped. Of the 14 initially selected candidate variables, 11 predictors (systolic blood pressure, heart rate, oxygen saturation, GCS, sex, age, HEMS vehicle type, whether the mission was located in a medical facility or nursing home, cardiac rhythm, time from emergency call to HEMS arrival and patient category) were included in the full model (Fig. 2). We assessed the significance of their effect on the outcome with the Wald test (Table 2). The odds ratios for the selected categorical variables are listed in Table 3. We allowed nonlinear effects by using restricted cubic splines for continuous variables (Fig 3).

**Table 2:** Univariate and multivariate Wald statistics for predictor variables

Variable		Univariate			Multivariate		
		χ^2^	d.f.	*P*-value	χ^2^	d.f.	*p*-value
Age		3082.7	3	<0.001	814.3	3	<0.001
Heart rate		122.8	3	<0.001	79.4	3	<0.001
Systolic blood pressure		425.3	3	<0.001	107.7	3	<0.001
Oxygen saturation		3502.1	3	<0.001	242.9	3	<0.001
Time to HEMS arrival		80.5	3	<0.001	8.9	3	0.030
GCS		6827.7	2	<0.001	1759.2	2	<0.001
Cardiac rhythm: VF, VT, asystole, or PEA		6613.3	1	<0.001	350.8	1	<0.001
Mission located in medical facility or nursing home, yes		108.4	1	<0.001	0.7	1	0.408
HEMS vehicle, ground unit^a^		11.6	1	<0.001	0.6	1	0.444
Sex, male		39.7	1	<0.001	1.0	1	0.325
Patient category	Trauma	21.0	1	<0.001	1.5	1	0.220
	Cardiac arrest	871.8	1	<0.001	7.4	1	0.007
	Neurological^b^	38.9	1	<0.001	10.9	1	<0.001
	Psychiatric or intoxication	35.7	1	<0.001	120.9	1	<0.001
	Other	34.8	1	<0.001	1.8	1	0.185
	Stroke	264.7	1	<0.001	13.7	1	<0.001
	Respiratory failure	70.2	1	<0.001	0.2	1	0.664
	Chest pain	1.8	1	0.186	0.0	1	0.876

*HEMS* Helicopter emergency medical services, *PEA* Pulseless electrical activity, *VF* Ventricular fibrillation, *VT* Ventricular tachycardia

^a^Rapid response vehicle + other

^b^Other than stroke

**Table 3:** Univariate and multivariate odds ratios for selected categorical predictor variables for the full model

Variable		Univariate OR (95% CI)	Multivariate OR (95% CI)
Cardiac rhythm: VF, VT, asystole, or PEA		29.33 (27.04 to 31.82)	3.90 (3.38 to 4.50)
Mission located in medical facility or nursing home, yes		1.68 (1.52 to 1.85)	1.06 (0.92 to 1.23)
HEMS vehicle, ground unit^a^		0.92 (0.87 to 0.96)	0.97 (0.88 to 1.06)
Sex, male		1.19 (1.12 to 1.25)	0.96 (0.88 to 1.04)
Patient category	Trauma	1.83 (1.41 to 2.37)	1.23 (0.88 to 1.71)
	Cardiac arrest	46.82 (36.27 to 60.44)	1.60 (1.14 to 2.24)
	Neurological^b^	2.32 (1.78 to 3.03)	0.57 (0.41 to 0.79)
	Psychiatric or intoxication	0.35 (0.25 to 0.49)	0.10 (0.07 to 0.15)
	Other	2.27 (1.73 to 2.98)	0.79 (0.56 to 1.12)
	Stroke	8.97 (6.89 to 11.69)	1.90 (1.35 to 2.66)
	Respiratory failure	3.29 (2.49 to 4.35)	1.08 (0.76 to 1.54)
	Chest pain	1.25 (0.90 to 1.75)	0.97 (0.65 to 1.45)
	Infection or Gynaecology and obstetrics	1.00 (reference)	1.00 (reference)

*HEMS* Helicopter emergency medical services, *PEA* Pulseless electrical activity, *VF* Ventricular fibrillation, *VT* Ventricular tachycardia

^a^Rapid response vehicle + other

^b^Other than stroke

**Fig. 4:**
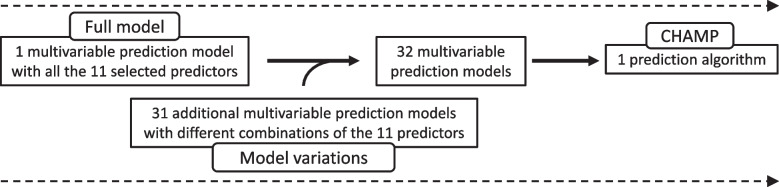
Development of the CHAMP. The model variations include six to eleven predictors per model, with one or more of the five predictors with the most missing data excluded. CHAMP, Critical HEMS Algorithm for Mortality Prediction

As we identified a notable amount of missing data for some variables (see Additional file 3: Table S2), we created 31 additional variations of the model to allow case-by-case exclusion of different combinations of the five variables with the most missing data: systolic blood pressure, heart rate, oxygen saturation, GCS and cardiac rhythm (see Additional file 4). They are designed to be applied whenever missing data for these variables are encountered. Combined, the 32 models form the CHAMP algorithm (Fig. 4). The CHAMP algorithm chooses the most suitable model for each patient depending on the available predictor variables. For example, if data for heart rate were missing, the algorithm would use the model that does not utilise heart rate as a predictor variable. The CHAMP algorithm can be accessed and the 30-day mortality estimate calculated using a calculator software designed for this purpose [32].

### Model performance, sensitivity analysis and internal validation

**Fig. 5:**
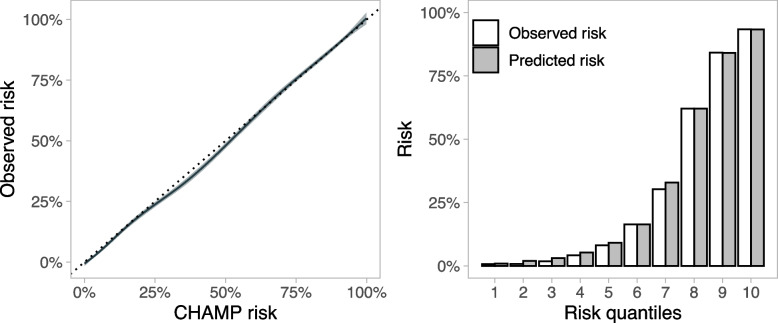
Calibration of the CHAMP (Critical HEMS Algorithm for Mortality Prediction) algorithm (solid line) with 95% confidence interval (shaded grey area) in the original nonimputed study population. The line was fitted using the generalised additive model (GAM). The dashed line represents ideal calibration

All the performance measures and optimism corrections based on internal validation for the CHAMP algorithm are presented in Table 4 and Fig. 5. For individual models, we observed AUROCs ranging from 0.868 to 0.927 and Brier scores from 0.125 to 0.093, depending on the excluded variables. Calibration intercepts were between -0.003 and 0.000, and slopes between 0.996 and 0.999 (see Additional file 5). The results of the sensitivity analysis excluding cardiac arrest patients differed from those of the primary analysis (Table 4).

**Table 4:** Performance and internal validation of the CHAMP algorithm along with the results of a sensitivity analysis without cardiac arrest patients

Metric	Algorithm performance			Validation performance			Sensitivity analysis		
	Estimate	95% CI		Optimism	95% CI		Estimate	95% CI	
		Lower	Upper		Lower	Upper		Lower	Upper
AUROC	0.932	0.929	0.935	0.011	0.008	0.015	0.887	0.881	0.893
Brier	0.091	0.089	0.093	-0.006	-0.008	-0.003	0.088	0.086	0.091
Slope	1.054	1.031	1.078	0.054	0.052	0.056	1.049	1.016	1.083
Intercept	-0.055	-0.094	-0.015	-0.055	-0.057	-0.052	-0.066	-0.121	-0.010

*AUROC* Area under the receiver operating characteristic

## Discussion

### Key findings

We analyzed data from 30,186 patients encountered by P-HEMS units, revealing a 30-day mortality rate of 30%. Notably, a substantial proportion of patients had missing data for predictor variables, as is often the case with pre-hospital data. After selecting and evaluating predictor variables, we developed a total of 32 prediction models. These models were then combined to form the Critical HEMS Algorithm for Mortality Prediction (CHAMP). With CHAMP, 30-day mortality in patients encountered by P-HEMS can be estimated using 11 easily obtainable variables.

For the full model with all the 11 variables, the analysis revealed that cardiac rhythms VF, VT, asystole, or PEA, indicated higher mortality risk. Mission location and time to HEMS arrival initially showed association with mortality risk, but these diminished in multivariate analysis. Type of HEMS vehicle and patient sex demonstrated weaker associations with mortality. Patient categories exhibited varying associations, with cardiac arrest and stroke indicating the highest mortality risk. Mortality increased with age, extreme systolic blood pressure values, and decreasing heart rate, oxygen saturation, and Glasgow Coma Scale (GCS) scores.

Following internal validation, we observed a promising preliminary performance with excellent discrimination and calibration. The sensitivity analysis without cardiac arrest patients revealed that the model exhibited slight variation but still performed acceptably. If our algorithm is externally validated, it can be used to calculate SMR in the patient population encountered by P-HEMS and possibly other EMS units and would offer a mortality estimation of patients based on initial assessment independent of pre-hospital interventions. To improve the algorithm’s accessibility, we developed a calculator software that can be accessed online [32].

### Interpretation

Seymour et al. studied the ability of pre-hospital factors easily obtainable at the scene to predict development of critical illness, defined as severe sepsis, delivery of mechanical ventilation or death at any point during hospitalization [33]. A development cohort consisted of patients encountered in Washington, USA, by either basic or advanced life support trained EMS and included neither physician-staffed ground EMS nor HEMS units, thus differing from our study setting. Patients with trauma or cardiac arrest were excluded, both of whom form a substantial proportion of patients treated by many HEMS systems. Based on their findings, a score was created to calculate the risk for critical illness, including patient sex, age, respiratory rate, oxygen saturation, systolic blood pressure, heart rate, GCS and nursing home location as predictors, many of which we found to have predictive value in our study. Seymour et al. reported a promising performance in internal validation with an AUROC of 0.77 and a Brier score of 0.04. However, the model’s applicability to HEMS systems may be of limited value, for the reasons discussed. The model’s discrimination was assessed by Kievlan et al. in a 2016 external validation study that reported an AUROC of 0.73 [34]. The model was further validated externally in the Dutch EMS system, achieving an AUROC of 0.74 [35]. In contrast to the original study and the previous external validation, the Dutch cohort included P-HEMS missions, although these covered only a small proportion (0.7%,* n*=22) of all patient encounters. We believe that the model proposed by Seymour et al. could be used in parallel with ours, as it serves to predict the need for intensive care, whereas our model focuses on mortality.

As Seymour et al. pointed out, their model is meant as a triage tool to be applied at the scene and needs to be simple. As our algorithm is not intended to be calculated at the scene, simplicity was not our priority, which allowed us to create a more complex model while still using obtainable variables.

The studied variables comply with the reporting policies equally agreed upon within the HEMS and EMS communities [22–24, 36]. The algorithm’s ability to variate according to different combinations of missing data enables its utility in the statistically challenging pre-hospital field where imperfections in data collection and availability are unavoidable. Due to the very nature of pre-hospital critical emergency medicine, certain physiological measures will not be achievable in every mission, even with best practices. For example, cardiac arrest patients, who constitute a major patient population for most HEMS teams, present with vital functions lacking, and some physiological parameters, such as oxygen saturation, thus being unmeasurable. In addition, pre-hospital settings often involve dynamic and unpredictable situations, and data collection may not always be feasible or prioritized amid the urgency of patient care. P-HEMS teams frequently operate with limited resources. Technological issues such as device malfunctions, connectivity problems, or user interface difficulties can contribute to missing data.

### Implications

It is crucial to perceive that CHAMP is not intended to provide prognostication for individual patients but rather to describe demographics of a group. For epidemiological research, it may be used to risk stratify a population of interest or to match the baseline characteristics of a control arm to those of an intervention arm, for example. SMR is the ratio of observed to predicted mortality. Predicted mortality, in turn, can be estimated with CHAMP. Using SMR as a performance measure enables benchmarking, quality assurance and prioritising targets for improvement.

Alongside external validation, another focus of future research should be the CHAMP algorithm’s conformity to changing registration policies and adaptation to future innovations as new clinical predictors and measurement methods are identified and adopted for pre-hospital critical emergency medicine.

### Strengths and limitations

We note several study strengths. The FHDB is large and includes data collected since 2012; data are collected systematically from multiple units. The HEMS units contributing to the database serve the whole of Finland and are an integral part of the national publicly funded healthcare system. The study sample included every P-HEMS mission in Finland during the study period. Our study has some limitations. P-HEMS missions constitute only a small proportion of all pre-hospital patient encounters. Although the CHAMP algorithm is designed to be used for patients treated by P-HEMS, some selection bias is possible, since the criteria for P-HEMS activation in Finland may vary from those in different health care systems.

A sensitivity analysis without cardiac arrest patients showed inconsistency in the results, most distinctly with respect to discrimination performance, suggesting that the applicability of the algorithm might be limited in settings with a divergent incidence of cardiac arrest. However, cardiac arrest patients form a substantial proportion of the patients treated by most P-HEMS [37–39].

We identified a high proportion of missing data and excluded variables with more than one-third of the data missing. To allow multiple imputation for the remaining variables, we assumed that the data were missing at random, which may be debated, but this bias may be reduced as the algorithm selects a model that accounts for some of the missing variables. The data were collected and entered manually into the database, which may have resulted in erroneous measurement and registration in addition to problems with inter-rater reliability. Nevertheless, the reliability of the FHDB has recently been evaluated and found to be acceptable for data registration [40].

## Conclusions

Based on a comprehensive and systematically gathered database, we developed and internally validated a novel prediction algorithm for 30-day mortality prediction in patients encountered by a P-HEMS unit. The algorithm combines 32 prediction models using 11 easily obtainable variables: systolic blood pressure, heart rate, oxygen saturation, GCS, sex, age, HEMS vehicle type, whether the mission was located in a medical facility or nursing home, cardiac rhythm, time to HEMS arrival and patient category according to dispatch code. If the current algorithm in time proves successful in external validation, it could be a useful research and quality assurance tool.

### Supplementary Information

**Additional file 1.** Locations and catchment areas of the FinnHEMS units.

**Additional file 2.** List of all FHDB variables.

**Additional file 3:** **Table S1.** Cumulative number of missing variables per encounter. **Table S2.** Missing data for each analysed variable.

**Additional file 4.** Estimates for each individual model.

**Additional file 5.** Performance measures for each individual model.

## Data Availability

All data are available upon reasonable request and may be obtained by contacting the corresponding author. The CHAMP calculator tool can be accessed on https://github.com/laamit/champCalculator.

## Abbreviations

AFib: Atrial fibrillation
AFlut: Atrial flutter
APACHE: Acute Physiology and Chronic Health Evaluation
AUROC: Area under the receiver operating characteristic
AV: Atrioventricular
CHAMP: Critical HEMS Algorithm for Mortality Prediction
CI: Confidence interval
EMS: Emergency medical services
EWS: Early warning score
FHDB: FinnHEMS database
GAM: Generalized additive model
GCS: Glasgow Coma Scale
HEMS: Helicopter emergency medical services
ICU: Intensive care unit
IQR: Interquartile range
MPM: Mortality Prediction Model
OR: Odds ratio
P-HEMS: Physician-staffed helicopter emergency medical services
PEA: Pulseless electrical activity
SAPS: Simplified Acute Physiology Score
SMR: Standardized mortality ratio
SVES: Supraventricular extrasystole
TRIPOD: Transparent Reporting of a multivariable prediction model for Individual; Prognosis Or Diagnosis
VES: Ventricular extrasystole
VF: Ventricular fibrillation
VT: Ventricular tachycardia

## References

[1] Knaus WA, Zimmerman JE, Wagner DP, Draper EA, Lawrence DE (1981). APACHE-acute physiology and chronic health evaluation: a physiologically based classification system. Crit Care Med.

[2] Bouch DC, Thompson JP (2008). Severity scoring systems in the critically ill. ContinEduc Anaesth Crit Care Pain.

[3] Vincent JL, Moreno R (2010). Clinical review: scoring systems in the critically ill. Crit Care.

[4] Jeong S (2018). Scoring Systems for the Patients of Intensive Care Unit. Acute Crit Care..

[5] Vincent JL, Moreno R, Takala J (1996). The SOFA (Sepsis-related Organ Failure Assessment) score to describe organ dysfunction/failure. On behalf of the Working Group on Sepsis-Related Problems of the European Society of Intensive Care Medicine. Intensive Care Med.

[6] Breslow MJ, Badawi O (2012). Severity scoring in the critically ill: part 1–interpretation and accuracy of outcome prediction scoring systems. Chest.

[7] Knaus WA, Draper EA, Wagner DP, Zimmerman JE (1985). APACHE II: a severity of disease classification system. Crit Care Med.

[8] Frankema SP, Ringburg AN, Steyerberg EW, Edwards MJ, Schipper IB, van Vugt AB (2004). Beneficial effect of helicopter emergency medical services on survival of severely injured patients. Br J Surg.

[9] Phipps MS, Cronin CA (2020). Management of acute ischemic stroke. BMJ.

[10] Maddock A, Corfield AR, Donald MJ, Lyon RM, Sinclair N, Fitzpatrick D (2020). Prehospital critical care is associated with increased survival in adult trauma patients in Scotland. Emerg Med J.

[11] Mathew TP, Menown IB, McCarty D, Gracey H, Hill L, Adgey AA (2003). Impact of pre-hospital care in patients with acute myocardial infarction compared with those first managed in-hospital. Eur Heart J.

[12] Goto Y, Funada A, Goto Y (2019). Impact of prehospital physician-led cardiopulmonary resuscitation on neurologically intact survival after out-of-hospital cardiac arrest: A nationwide population-based observational study. Resuscitation..

[13] Martin-Rodriguez F, Lopez-Izquierdo R, Del Pozo Vegas C (2020). Can the prehospital National Early Warning Score 2 identify patients at risk of in-hospital early mortality? A prospective, multicenter cohort study. Heart Lung.

[14] Martin-Rodriguez F, Castro-Villamor MA, Del Pozo Vegas C (2019). Analysis of the early warning score to detect critical or high-risk patients in the prehospital setting. Intern Emerg Med.

[15] Williams TA, Tohira H, Finn J, Perkins GD, Ho KM (2016). The ability of early warning scores (EWS) to detect critical illness in the prehospital setting: a systematic review. Resuscitation.

[16] Grasner JT, Meybohm P, Lefering R (2011). ROSC after cardiac arrest–the RACA score to predict outcome after out-of-hospital cardiac arrest. Eur Heart J.

[17] Patel R, Nugawela MD, Edwards HB (2018). Can early warning scores identify deteriorating patients in pre-hospital settings? A systematic review. Resuscitation.

[18] Hoikka M, Silfvast T, Ala-Kokko TI (2018). Does the prehospital National Early Warning Score predict the short-term mortality of unselected emergency patients?. Scand J Trauma Resusc Emerg Med.

[19] Pappinen J, Olkinuora A, Laukkanen-Nevala P (2019). Defining a mission-based method to determine a HEMS unit's actual service area. Scand J Trauma Resusc Emerg Med.

[20] Saviluoto A, Bjorkman J, Olkinuora A (2020). The first seven years of nationally organized helicopter emergency medical services in Finland - the data from quality registry. Scand J Trauma Resusc Emerg Med.

[21] Collins G, Reitsma J, Altman D, Moons K (2015). Transparent Reporting of a multivariable prediction model for Individual Prognosis Or Diagnosis (TRIPOD): the TRIPOD Statement. BMC Med.

[22] Kruger AJ, Lockey D, Kurola J (2011). A consensus-based template for documenting and reporting in physician-staffed pre-hospital services. Scand J Trauma Resusc Emerg Med.

[23] Perkins GD, Jacobs IG, Nadkarni VM (2015). Cardiac arrest and cardiopulmonary resuscitation outcome reports: update of the Utstein Resuscitation Registry Templates for Out-of-Hospital Cardiac Arrest: a statement for healthcare professionals from a task force of the International Liaison Committee on Resuscitation (American Heart Association, European Resuscitation Council, Australian and New Zealand Council on Resuscitation, Heart and Stroke Foundation of Canada, InterAmerican Heart Foundation, Resuscitation Council of Southern Africa, Resuscitation Council of Asia); and the American Heart Association Emergency Cardiovascular Care Committee and the Council on Cardiopulmonary, Critical Care Perioperative and Resuscitation. Circulation.

[24] Sunde GA, Kottmann A, Heltne JK (2018). Standardised data reporting from pre-hospital advanced airway management – a nominal group technique update of the Utstein-style airway template. Scand J Trauma Resusc Emerg Med.

[25] van Buuren S, Groothuis-Oudshoorn K (2011). mice: Multivariate Imputation by Chained Equations in R. J Stat Softw.

[26] Ong ME, Shin SD, De Souza NN (2015). Outcomes for out-of-hospital cardiac arrests across 7 countries in Asia: The Pan Asian Resuscitation Outcomes Study (PAROS). Resuscitation..

[27] R Development Core Team. R: A Language and Environment for Statistical Computing. Vienna, Austria: R Foundation for Statistical Computing; 2021. https://www.R-project.org/. Accessed 8 Dec 2021.

[28] Harrel FE. rms: Regression Modeling Strategies. R package version 6.2-0 2021. https://CRAN.R-project.org/package=rms. Accessed 4 May 2021.

[29] Bartlett J. bootImpute: Bootstrap Inference for Multiple Imputation. R package version 1.2.0 2021. https://cran.r-project.org/package=bootImpute. Accessed 4 May 2021.

[30] Wickham H (2016). ggplot2: Elegant Graphics for Data Analysis.

[31] Sachs MC. plotROC: A Tool for Plotting ROC Curves J Stat Softw. 2017;79:2. 10.18637/jss.v079.c02 PMC6347406 30686944

[32] Lääperi M, Reitala E, Nuottonen O. The CHAMP Calculator. https://github.com/laamit/champCalculator. Accessed 11 April 2022.

[33] Seymour CW, Kahn JM, Cooke CR, Watkins TR, Heckbert SR, Rea TD (2010). Prediction of critical illness during out-of-hospital emergency care. JAMA.

[34] Kievlan DR, Martin-Gill C, Kahn JM (2016). External validation of a prehospital risk score for critical illness. Crit Care.

[35] Veldhuis LI, Hollmann MW, Kooij FO, Ridderikhof ML (2021). A pre-hospital risk score predicts critical illness in non-trauma patients transported by ambulance to a Dutch tertiary referral hospital. Scand J Trauma Resusc Emerg Med.

[36] Tonsager K, Kruger AJ, Ringdal KG, Rehn M, Group PETC (2020). Template for documenting and reporting data in physician-staffed pre-hospital services: a consensus-based update. Scand J Trauma Resusc Emerg Med..

[37] Alstrup K, Petersen JAK, Sollid S, Johnsen SP, Rognås L. Mortality and hospitalisation in the Danish Helicopter Emergency Medical Service (HEMS) population from 2014 to 2018: a national population-based study of HEMS triage. BMJ Open. 2020;10:e038718. https://bmjopen.bmj.com/content/10/8/e038718. Accessed 14 Sept 2021. 10.1136/bmjopen-2020-038718 PMC7462229 32868364

[38] Rzonca P, Galazkowski R, Panczyk M, Gotlib J (2018). Polish Helicopter Emergency Medical Service (HEMS) Response to Out-of-Hospital Cardiac Arrest (OHCA): A Retrospective Study. Med Sci Monit.

[39] Lyon RM, Nelson MJ (2013). Helicopter emergency medical services (HEMS) response to out-of-hospital cardiac arrest. Scand J Trauma Resusc Emerg Med.

[40] Heino A, Iirola T, Raatiniemi L (2019). The reliability and accuracy of operational system data in a nationwide helicopter emergency medical services mission database. BMC Emerg Med.

