# Supplementary material for: Development and internal validation of an algorithm for estimating mortality in patients encountered by physician-staffed helicopter emergency medical services
Source: Scand J Trauma Resusc Emerg Med. 2024 Apr 23;32:33. doi: 10.1186/s13049-024-01208-y (PMC11040883; doi:10.1186/s13049-024-01208-y)
Supplement: Supplementary file 1 — Additional file 1. Locations and catchment areas of the FinnHEMS units. [file 13049_2024_1208_MOESM1_ESM.docx]

Additional file 1
Locations and catchment areas of the FinnHEMS units


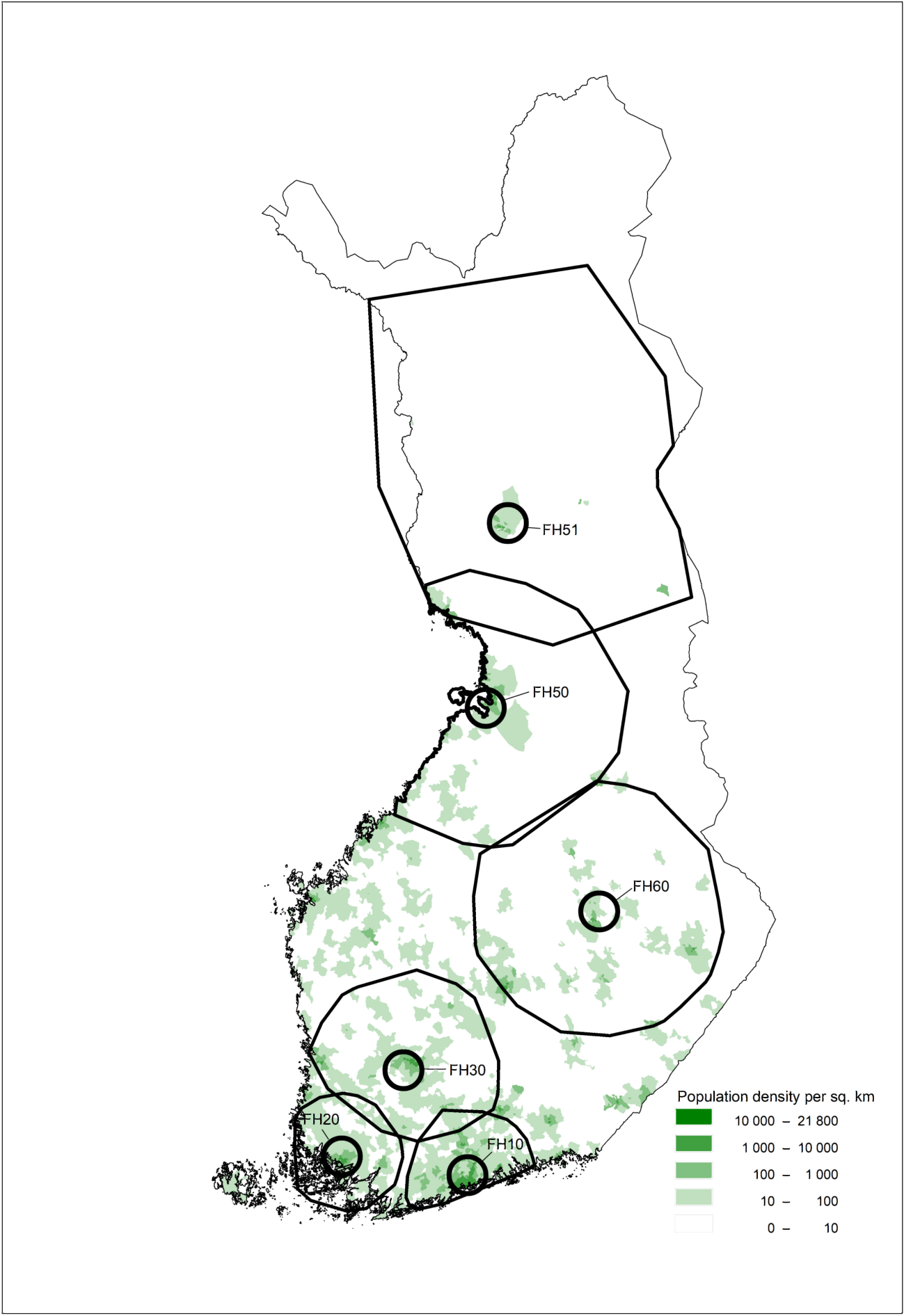


FH51 is a paramedic-only staffed unit not included in the study; the other units are physician-staffed. FH = FinnHEMS unit
