# Supplementary material for: Development and internal validation of an algorithm for estimating mortality in patients encountered by physician-staffed helicopter emergency medical services
Source: Scand J Trauma Resusc Emerg Med. 2024 Apr 23;32:33. doi: 10.1186/s13049-024-01208-y (PMC11040883; doi:10.1186/s13049-024-01208-y)
Supplement: Supplementary file 2 — Additional file 2. List of all FHDB variables. [file 13049_2024_1208_MOESM2_ESM.docx]

List of FHDB variables

The age and sex of a patient and the occurrence of death within 30 days were derived from the national resident register provided by Digital and Population Data Services Agency and matched with FHDB using patient ID.

| Variable |
| --- |
| Event operational descriptors |
| Alarm call time |
| Alarm Hospital District |
| Alarm ID |
| Alarm Medical Facility type |
| Alarm time |
| Ambulance |
| Ambulance alarm time |
| Ambulance time when at scene |
| Base |
| Bystander assisted resuscitation? |
| City |
| Country |
| Date |
| Delay: alarm to mobile |
| Delay: alarm to patient |
| Dispatch code |
| Dispathcer |
| Doctor |
| Final ROSC |
| First resonder time when at scene |
| First responder |
| First responder alarm time |
| Ground unit use effect on prognosis |
| HEMS Crew Member |
| HEMS score |
| Hour of alarm |
| Known limitations of treatment |
| Medical Facility? |
| Municipality of recidence |
| Number of patients |
| Origin of mission cancellation |
| Origin of mission cancellation (text) |
| Own transportation code |
| Own unit location at alarm time |
| Paramedic |
| Patient ID |
| Patient met enroute to hospital |
| Pilot |
| Reason for delay time |
| Reason for delay time (text) |
| Reason for ground unit use |
| Reason for mission cancel |
| ROSC? |
| Temporary ROSC |
| Time at hospital |
| Time at scene |
| Time avalable for new mission |
| Time mission ended |
| Time mobile |
| Time of cardiac arrest |
| Time of mission cancel |
| Time transport started |
| Time when at patient |
| Time when at scene |
| Total duration |
| Transport duration |
| Transportation code |
| Transporting unit |
| Type of treatment limitations |
| Vechicle registration number |
| Vechicle type |
|  |
| Patient descriptors |
| ASA class |
| Cardiogenic reason? |
| ECOG class |
| Final Heart rate |
| Final Systolic bloodpressure |
| Final end tidal CO2 |
| Final GCS eyes |
| Final GCS motor |
| Final GCS total |
| Final GCS verbal |
| Final pain |
| Final Respiratory rate |
| Final rhythm |
| Final saturation |
| Initial Heart rate |
| Initial Systolic blood pressure |
| Initial end tidal CO2 |
| Initial GCS eyes |
| Initial GCS motor |
| Initial GCS total |
| Initial GCS verbal |
| Initial pain |
| Initial Respiratory rate |
| Initial rhythm |
| Initial rhythm |
| Initial saturation |
| Main medical reason |
| Primary diagnosis |
| Primary ICPC code |
| Resuscitation commenced? |
| Secondary ICPC code |
| Secundary diagnosis |
| Type of trauma |
| Witnessed cardiac arrest? |
|  |
| Process mapping |
| Advanced control of haemorhage |
| Airway - Analgesia? |
| Airway - local anesthetic |
| Airway - medication? |
| Airway - Neuromucular Blocking Agent? |
| Airway - sedative? |
| Airway management |
| Analgesic |
| Antibiotic |
| Basic controll of haemorhage |
| Cardioversion |
| Central IV access |
| Chest tube |
| Defibrillation |
| Diagnostic measures |
| EKG |
| External pacing |
| Fibrinolytic therapy |
| Finger thoracostomy |
| Intraosseal access |
| invasive monitoring |
| Jaw thrust |
| Manual ventilation |
| Mechanical ventilation |
| Medication administered |
| Neromuscular Blocking Agent |
| No airway management |
| No circulatory management |
| Oropharyngeal airway |
| Other action |
| Other action (text) |
| Other actions |
| Other circulatory management |
| Other circulatory management (text) |
| Other diagnostic measure |
| Other diagnostic measure (text) |
| Other medication |
| Peripheral IV access |
| POC laboratory |
| POC ultrasound |
| Reponation of fracture |
| Sedative |
| Spinal immobilisation |
| Therapeutic hypothermia |
| Vasoactive drug |
|  |
| Quality indicators and mission outcome |
| Apiration/voimt during intubation |
| Blood pressure after intervention |
| Blood pressure at hospital |
| Bradycardia during intubation |
| Dental damage during intubation |
| End tidal CO2 after intervention |
| End tidal CO2 at hospital |
| Esophageal intubation |
| Failed intubation? |
| Heart rate after intervention |
| Heart rate at hospital |
| Hypotension during intubation |
| Hypoxia during intubation |
| Indication for airway management |
| Indication for airway management (text) |
| Initial respiratory rate |
| Initial blood pressure |
| Initial GCS eyes |
| Initial GCS motor |
| Initial GCS verbal |
| Initial heart rate |
| Initial saturation |
| Intubation success |
| Logistic benefit of helicopter transport |
| Main stem intubation |
| Other intubation complication |
| Oxygen? |
| Patient escorted by doctor? |
| Patient transported? |
| Peformer of airway management |
| Receiving hospital |
| Respiratory rate after intervention |
| Respiratory rate at hospital |
| Saturation after intervention |
| Saturation at hospital |
| Survival to hospital |
