# Supplementary material for: Development and internal validation of an algorithm for estimating mortality in patients encountered by physician-staffed helicopter emergency medical services
Source: Scand J Trauma Resusc Emerg Med. 2024 Apr 23;32:33. doi: 10.1186/s13049-024-01208-y (PMC11040883; doi:10.1186/s13049-024-01208-y)
Supplement: Supplementary file 3 — Additional file 3: Table S1. Cumulative number of missing variables per encounter. Table S2. Missing data for each analysed variable. [file 13049_2024_1208_MOESM3_ESM.docx]

**Table S1** The cumulative number of missing variables per encounter (comprising only the variables that were included in the final model)

| Number of missing variables | *n* | % | Cumulative % |
| --- | --- | --- | --- |
| 0 | 18 215 | 60,3% | 60,3% |
| 1 | 3164 | 10,5% | 70,8% |
| 2 | 1213 | 4,0% | 74,8% |
| 3 | 5281 | 17,5% | 92,3% |
| 4 | 650 | 2,2% | 94,5% |
| 5 | 1533 | 5,1% | 99,6% |
| 6 | 122 | 0,4% | 100,0% |
| 7 | 8 | 0,0% | 100,0% |

**Table S2** Missing data for each analysed variable

| Variable | Missing, n (% of all cases) |
| --- | --- |
| Age | 0 (0.0) |
| Heart rate | 8033 (26.6) |
| Systolic blood pressure | 8605 (28.5) |
| Respiratory rate | 13 530 (44.8) |
| Oxygen saturation | 9066 (30.0) |
| Time to HEMS arrival | 0 (0.0) |
| Time to EMS arrival | 19 642 (65.1) |
| GCS | 2198 (7.3) |
| Cardiac rhythm | 2413 (8.0) |
| Medical facility or nursing home | 0 (0.0) |
| HEMS vehicle | 0 (0.0) |
| Number of patients | 0 (0.0) |
| Patient sex | 354 (1.2) |
| Patient category | 0 (0.0) |
| Death within 30 days | 1817 (6.0) |

EMS, emergency medical services; GCS, Glasgow Coma Scale; HEMS, helicopter emergency medical services.
