# Supplementary material for: Development and internal validation of an algorithm for estimating mortality in patients encountered by physician-staffed helicopter emergency medical services
Source: Scand J Trauma Resusc Emerg Med. 2024 Apr 23;32:33. doi: 10.1186/s13049-024-01208-y (PMC11040883; doi:10.1186/s13049-024-01208-y)

Estimates for all model variations

**List of predictor variables, units, categories, and abbreviations in the data**

Age, years (age)
Heart rate, beats per minute (pulse)
Systolic blood pressure, mmHg (rr)
Oxygen saturation, % (spo2)
Time to HEMS arrival, minutes (time_from_alarm)
GCS, ordinal units (gcs)
Cardiac rhythm: VF, VT, asystole, or PEA (cardiac_rhythm)
Mission located in medical facility or nursing home (med_facility)
HEMS vehicle, ground unit* (vehicle_ground_unit)
Sex, male (sex_man)
Patient category:
 Trauma (code_trauma)
 Cardiac arrest (code_cardiac_arrest)
 Neurological** (code_neuro)
 Psychiatric or intoxication (code_psyc_intox)
 Other (code_other)
 Stroke (code_stroke)
 Respiratory failure (code_respitory)
 Chest pain (code_chest_pain)

GCS, Glasgow Coma Scale; HEMS, helicopter emergency medical services; PEA, pulseless electrical activity; VF, ventricular fibrillation; VT, ventricular tachycardia. *Rapid response vehicle + other; **Other than stroke.


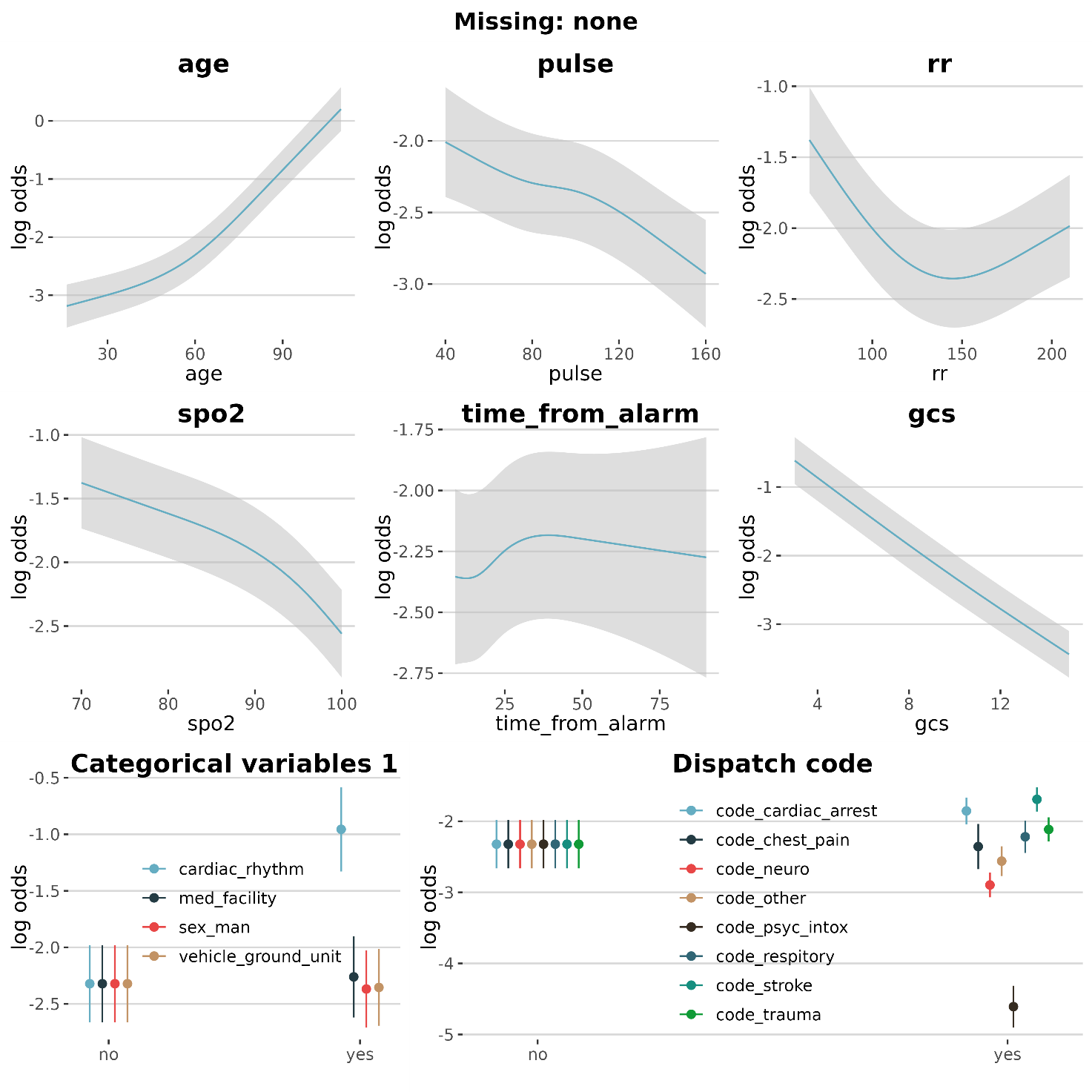


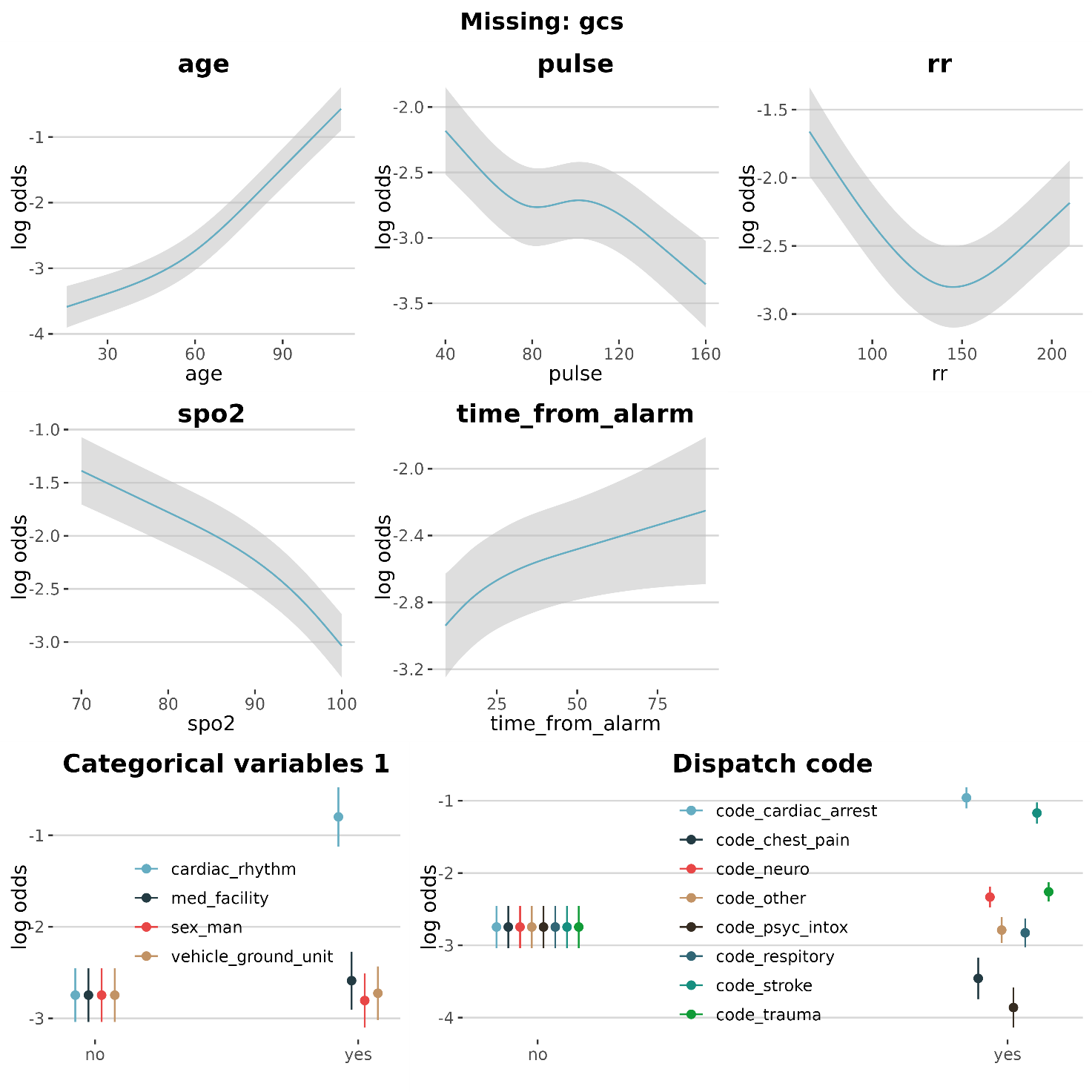

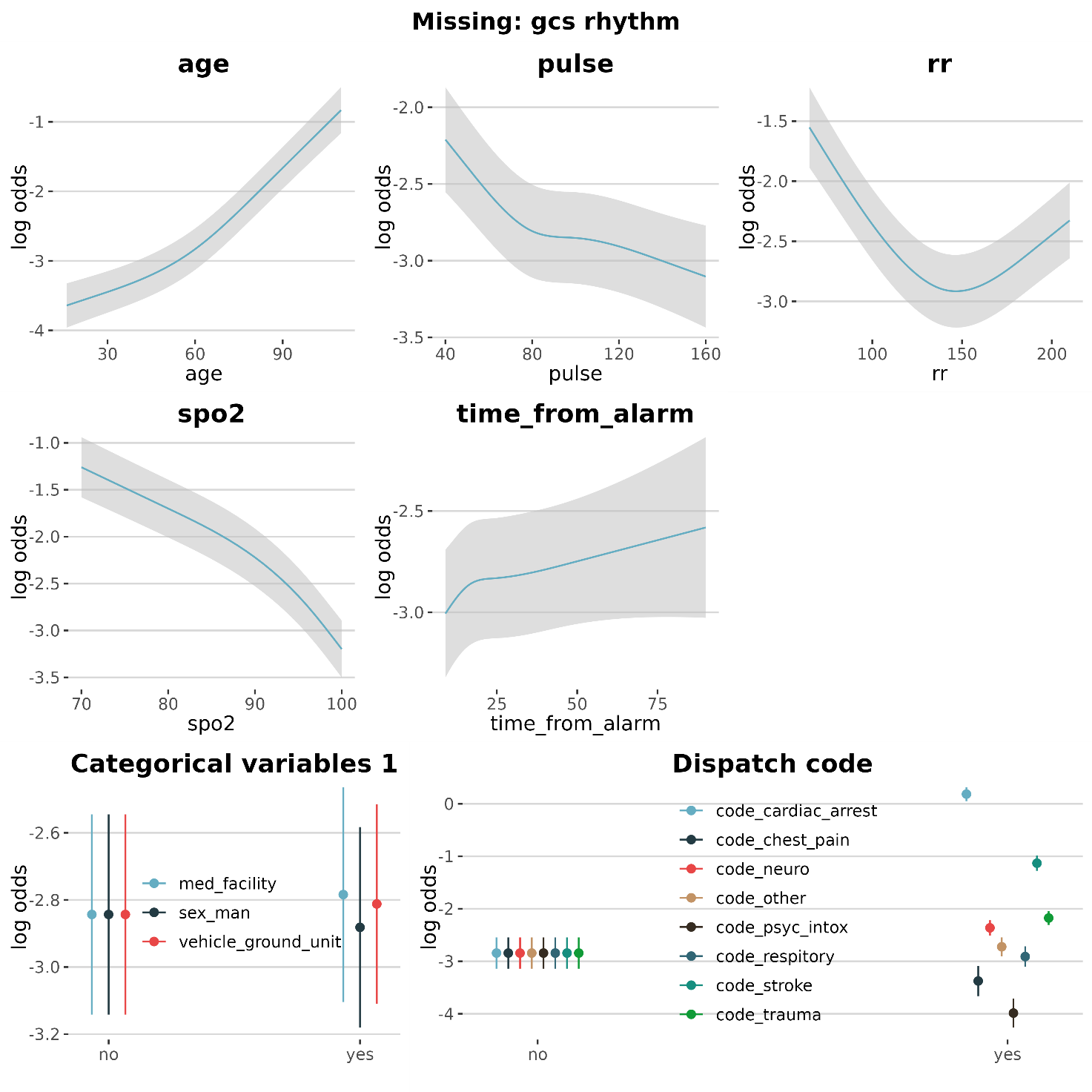

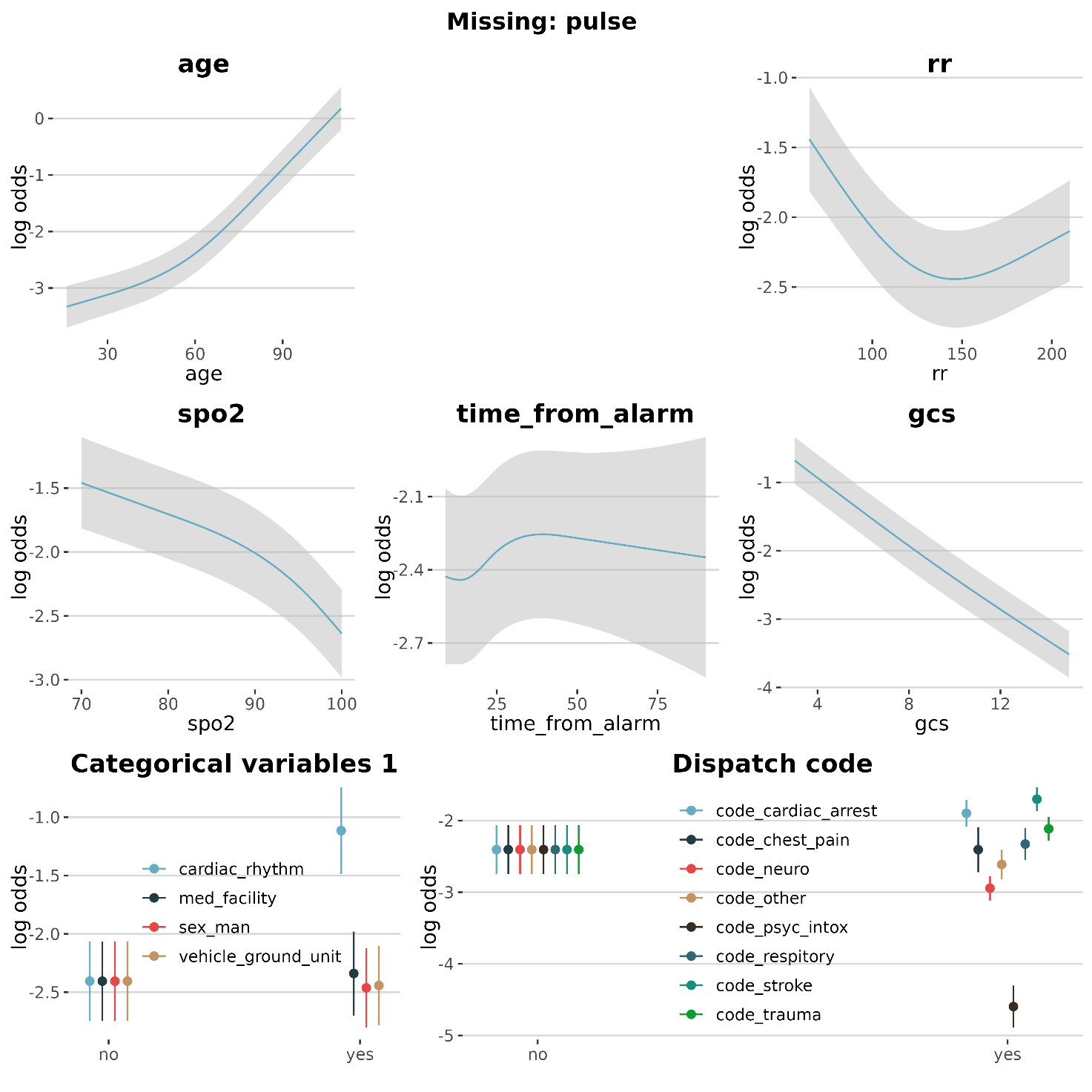

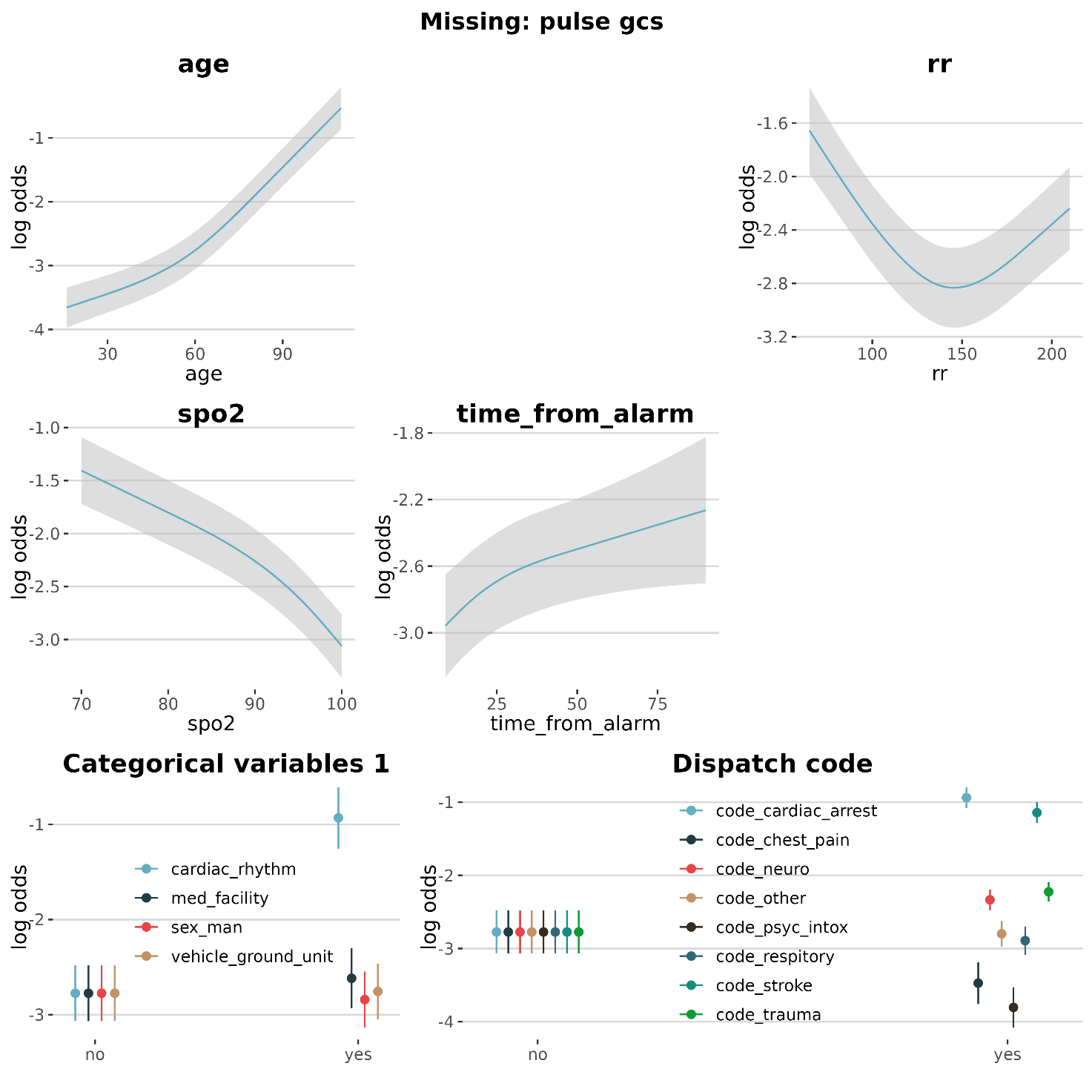

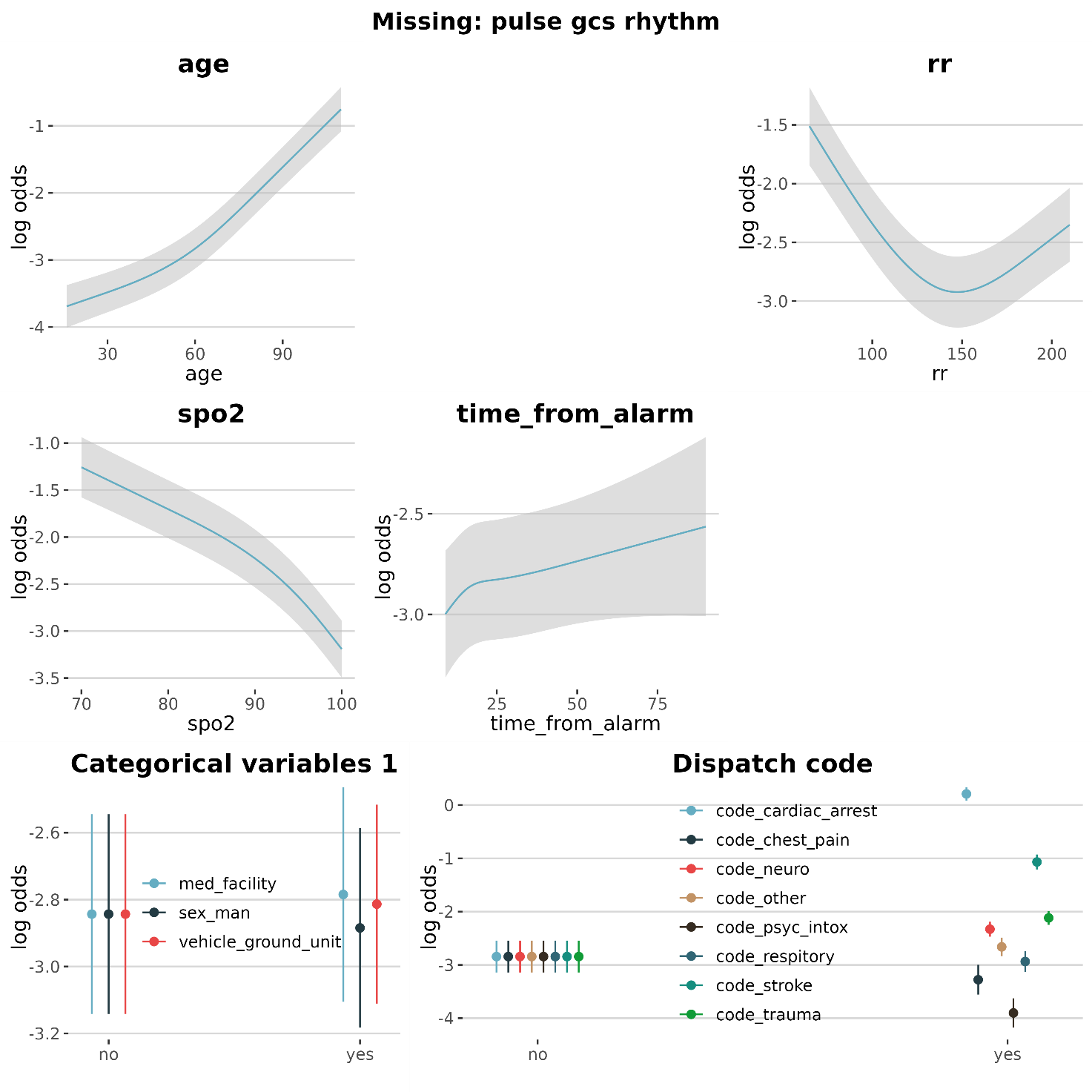

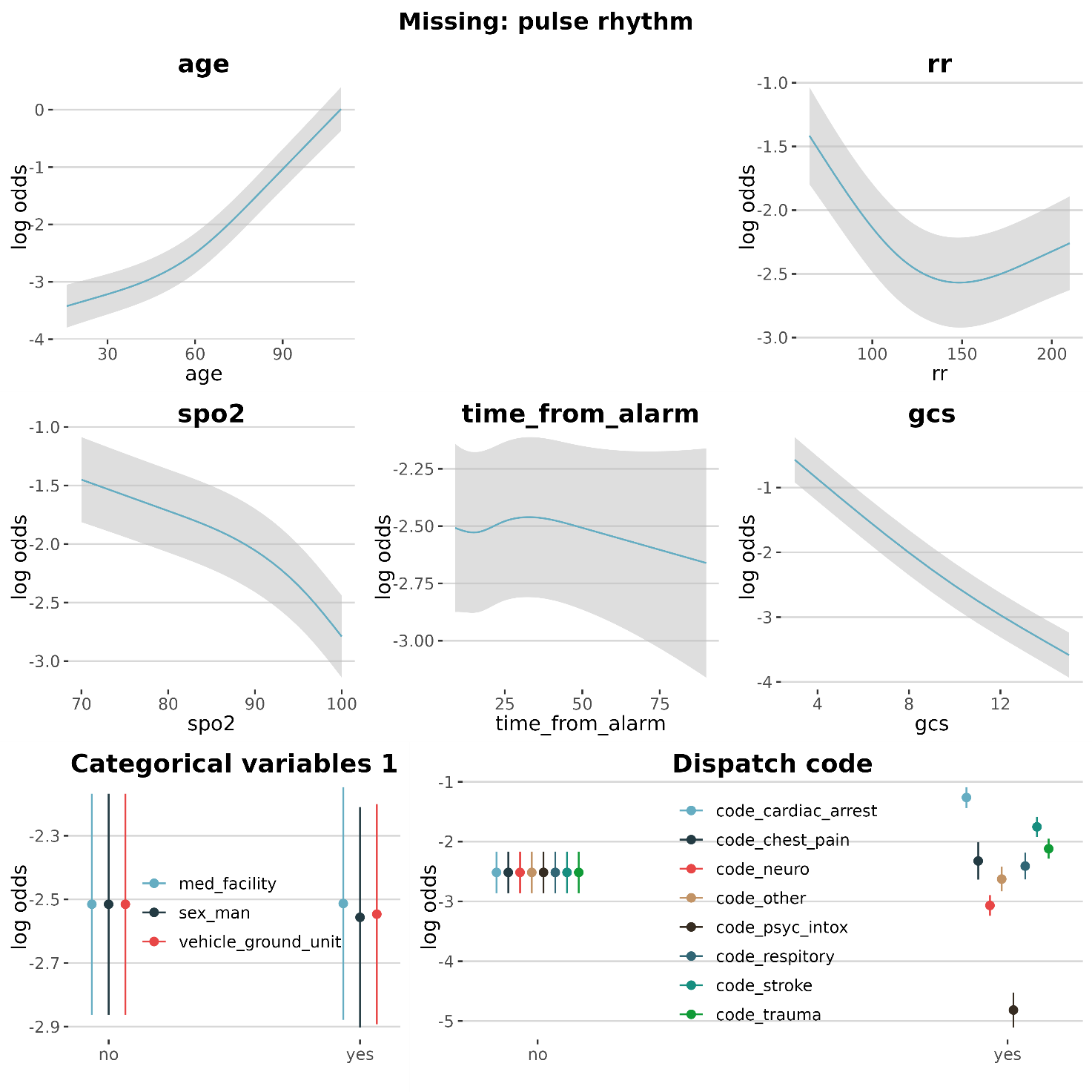

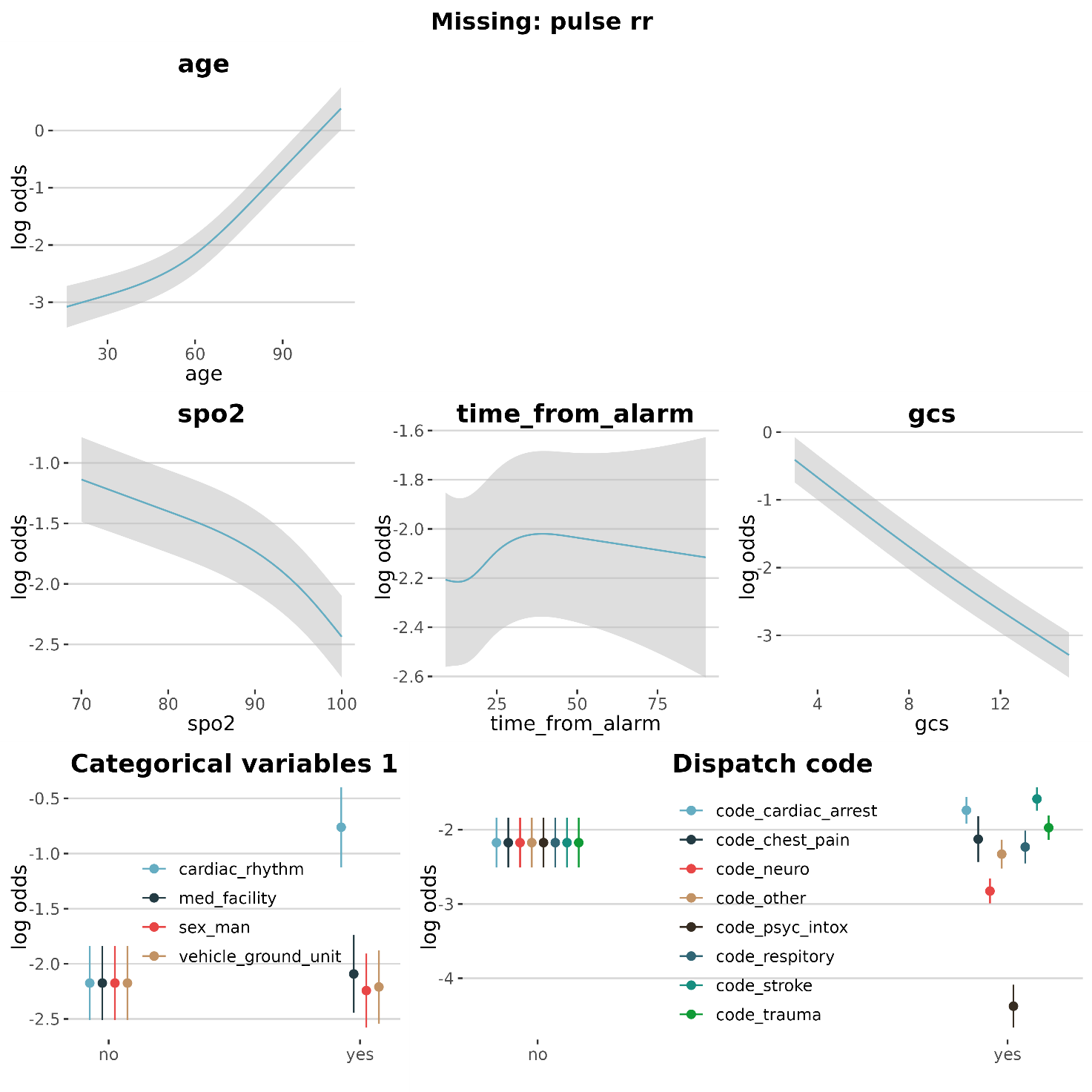

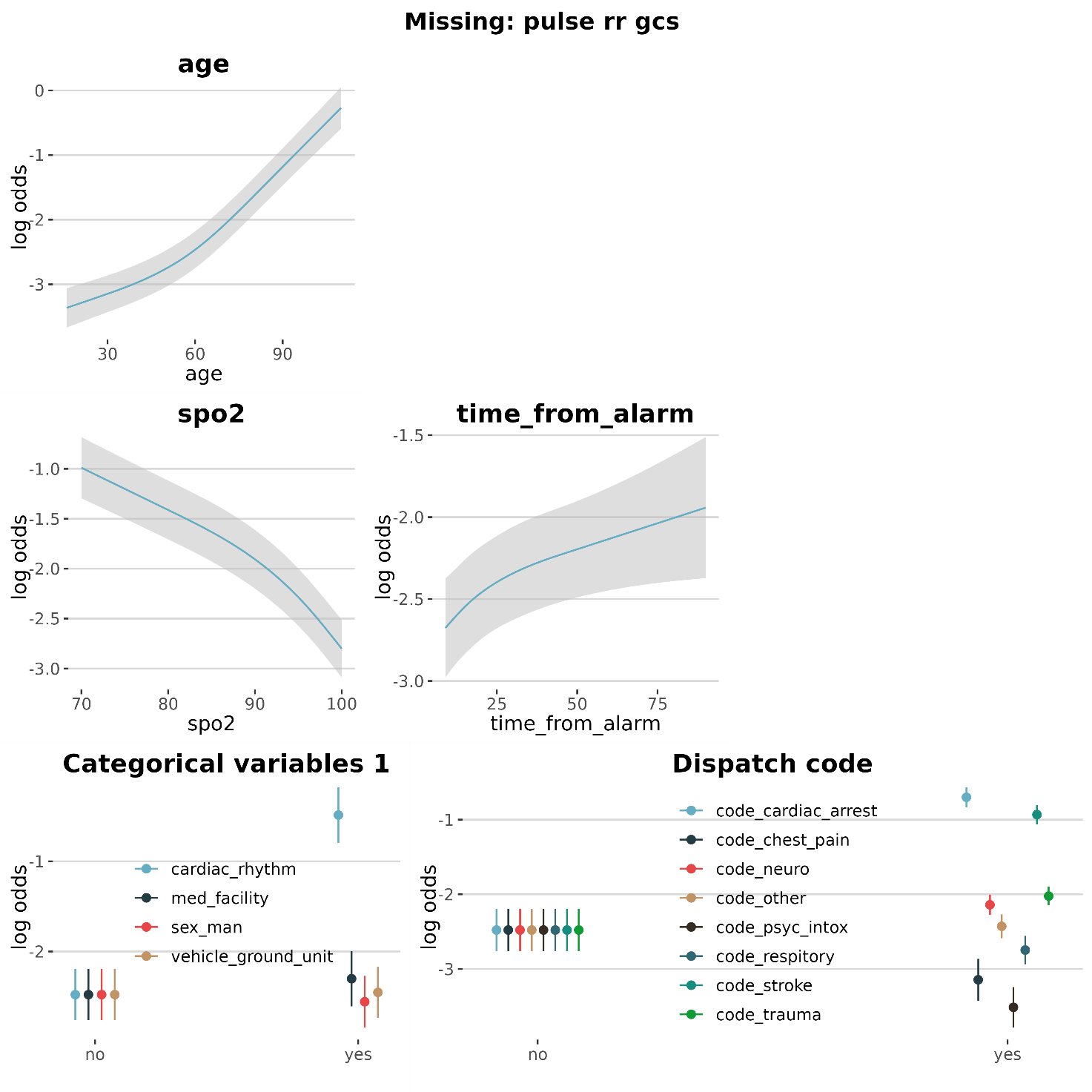

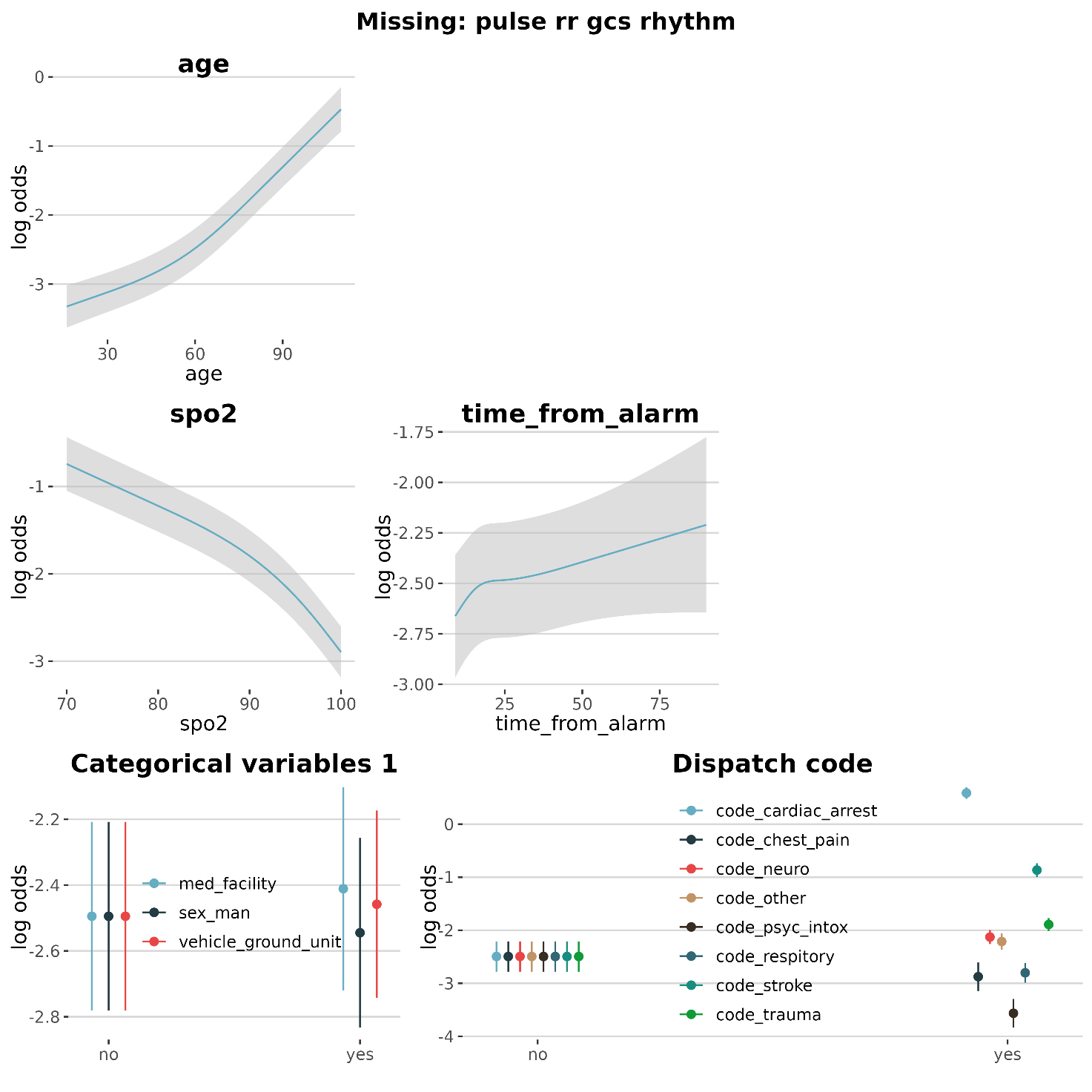

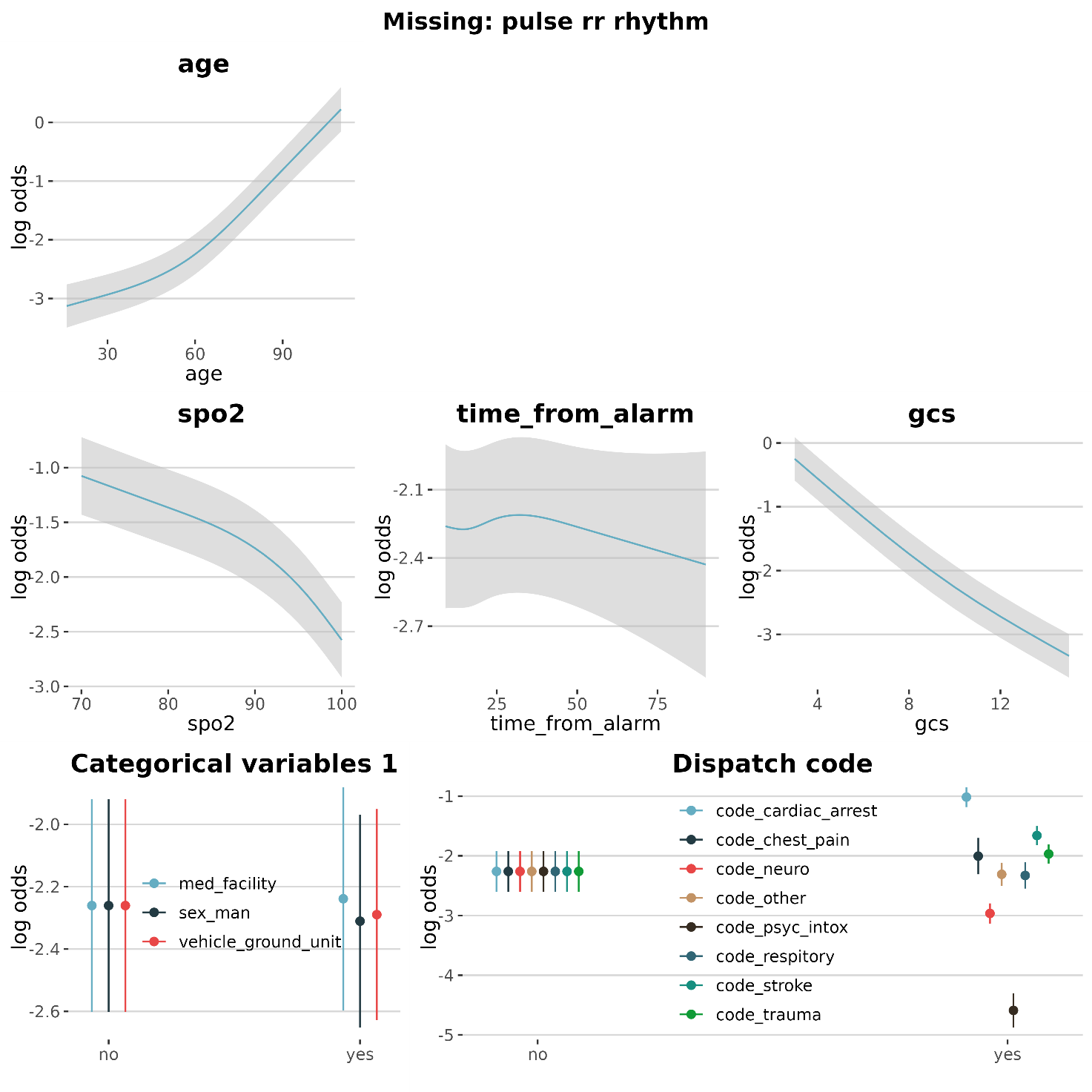

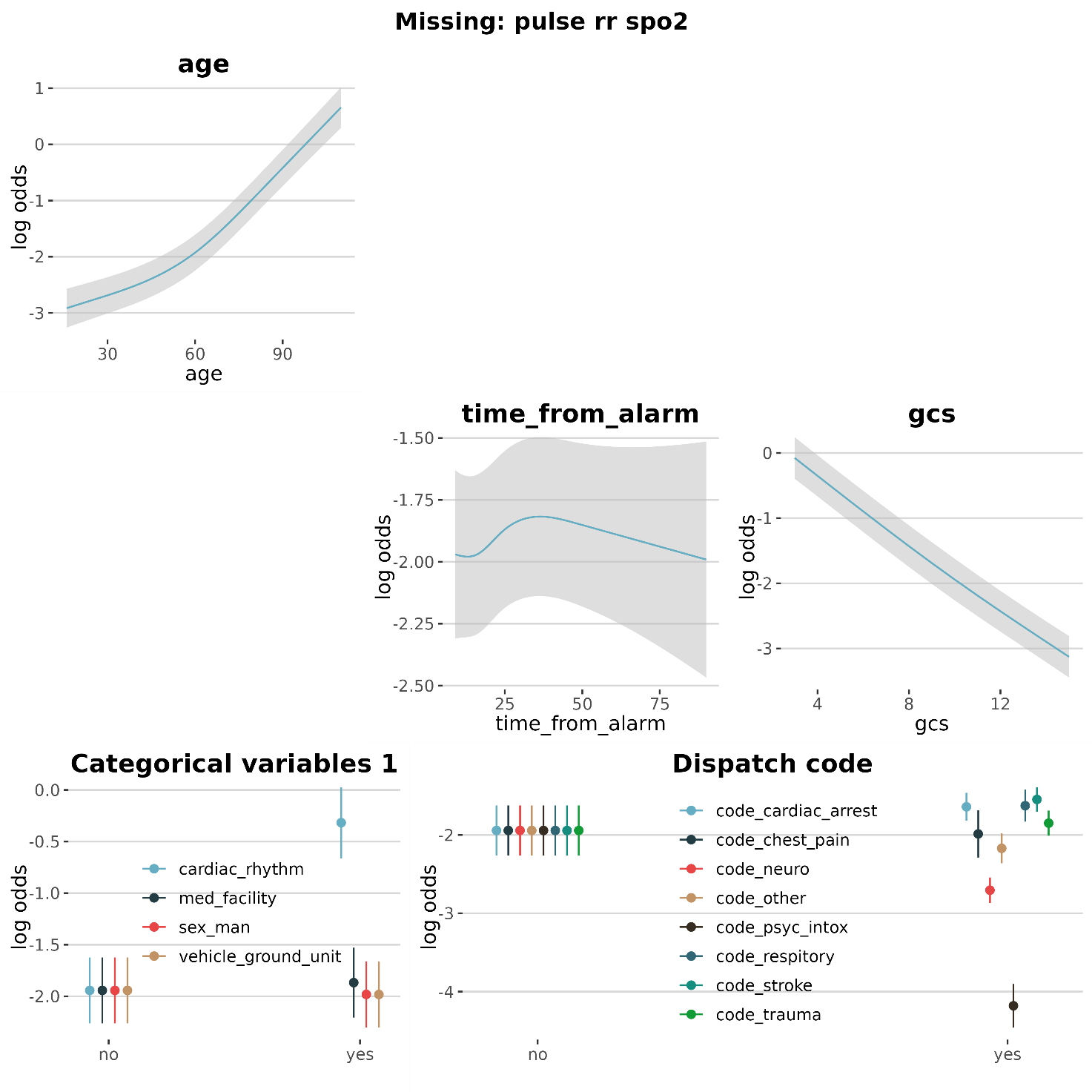

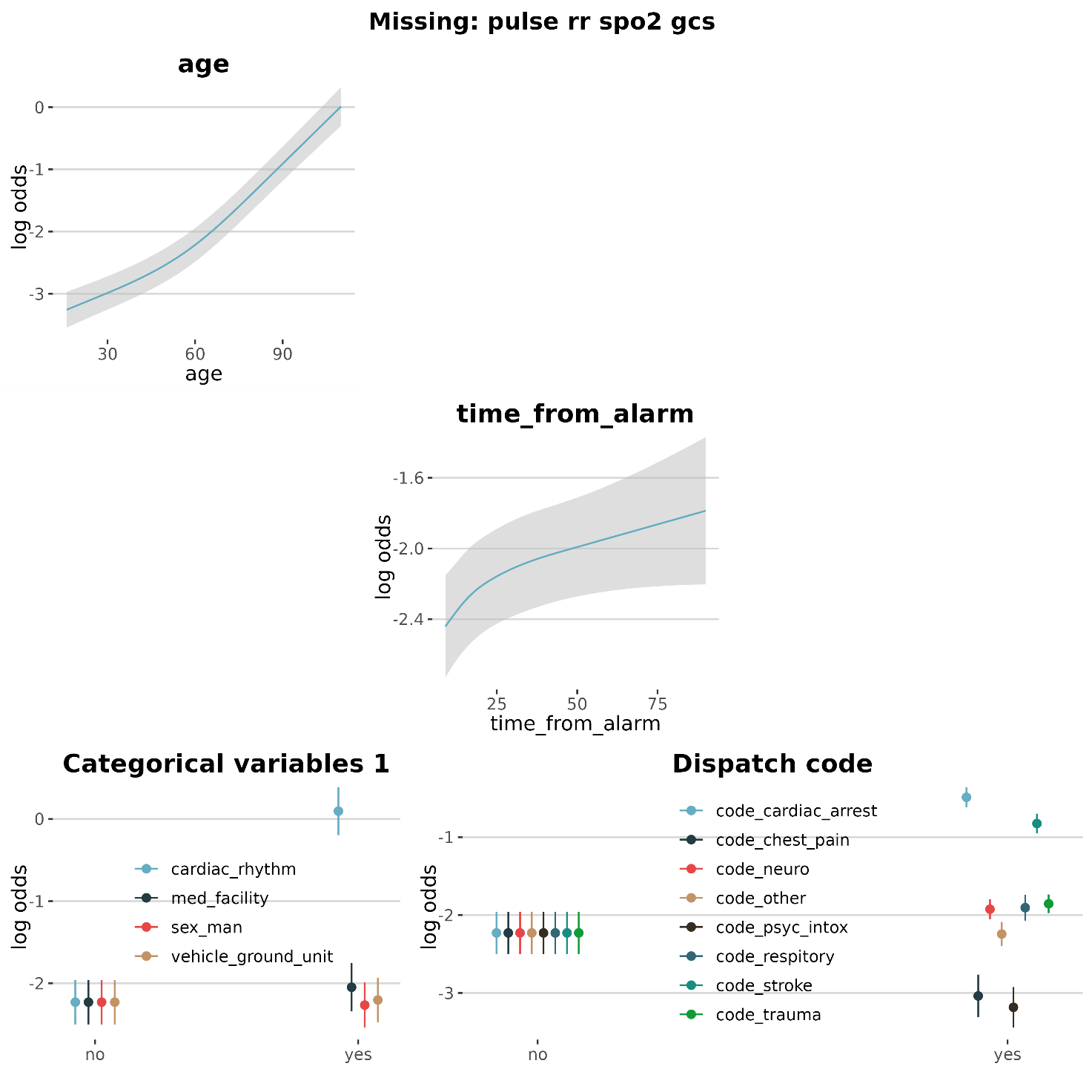

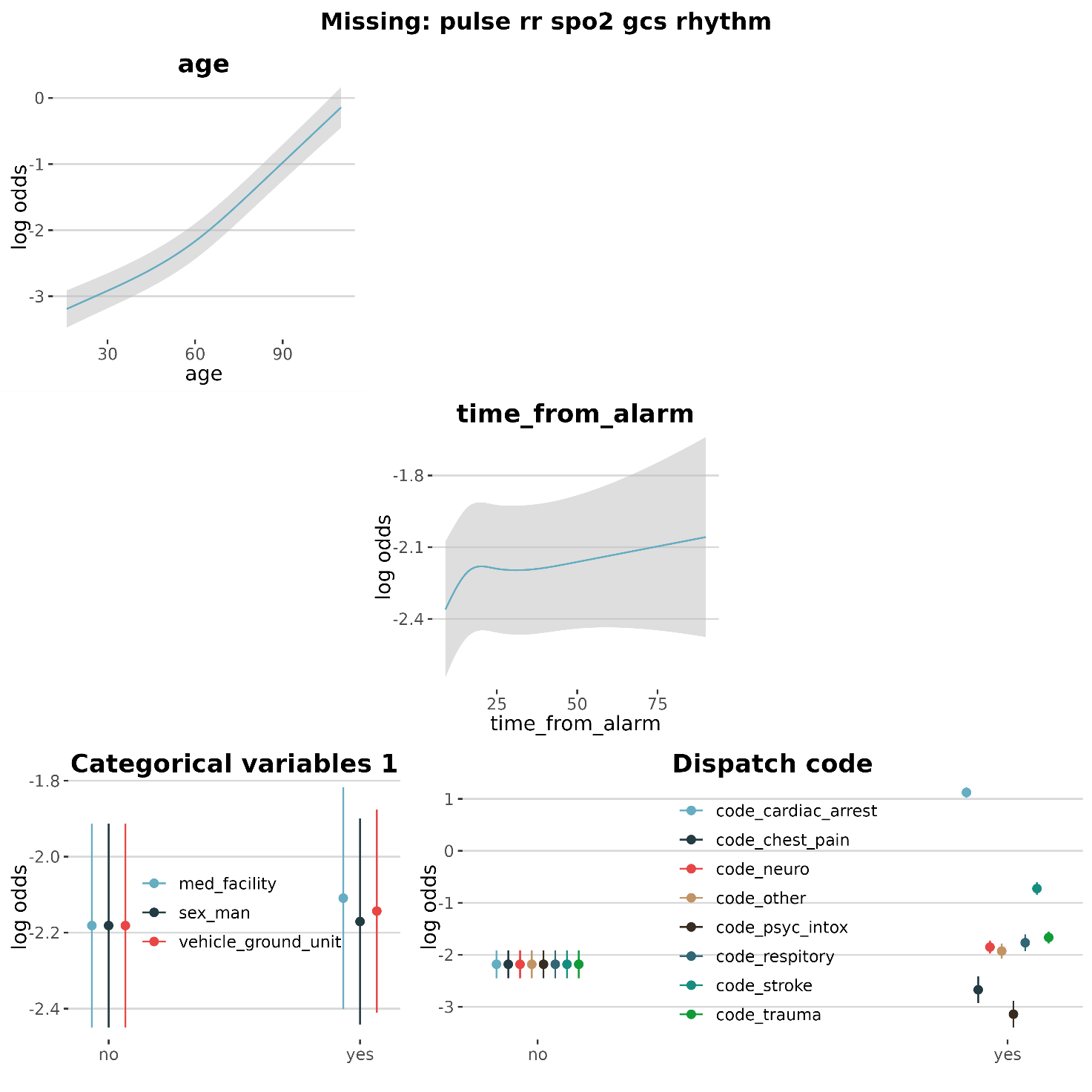

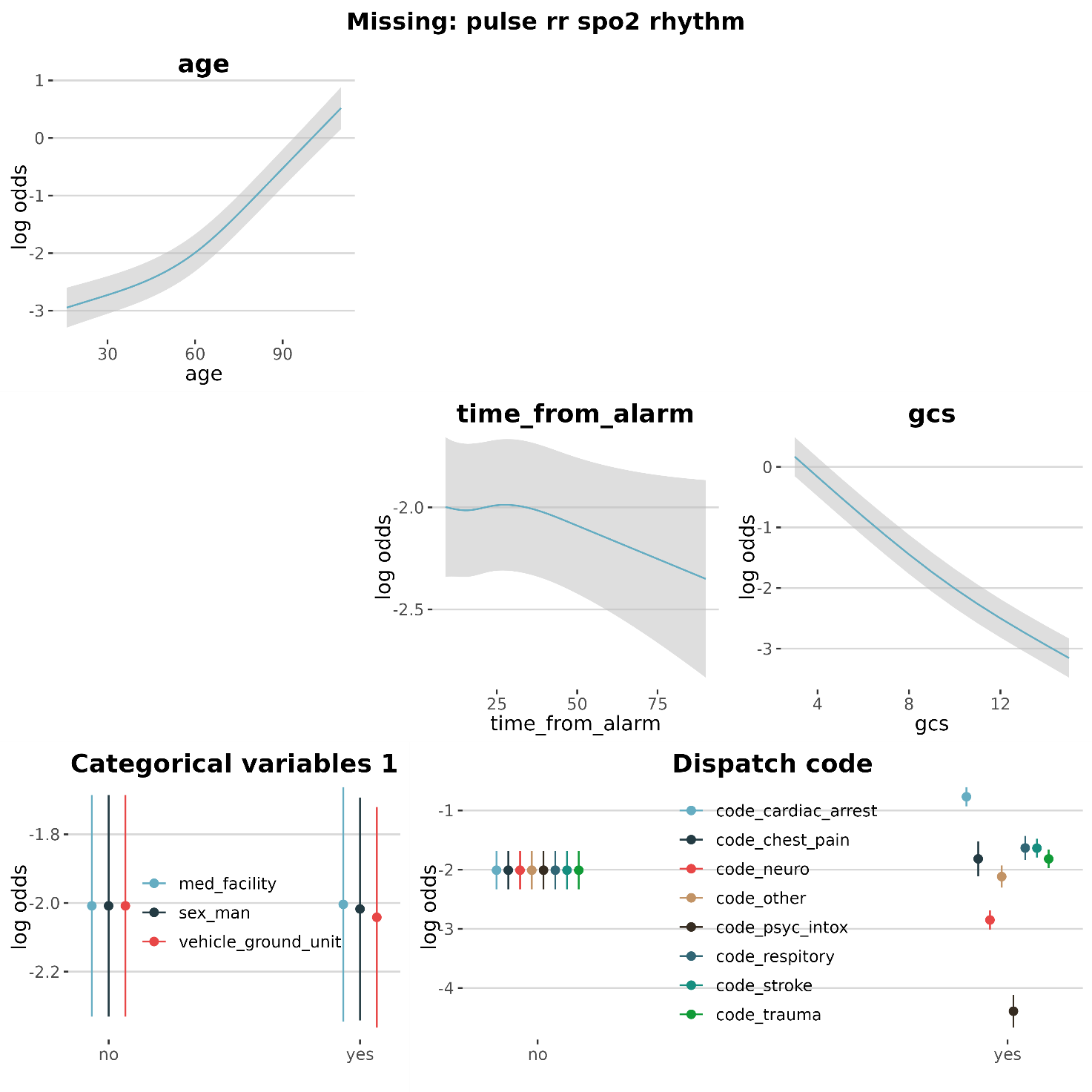

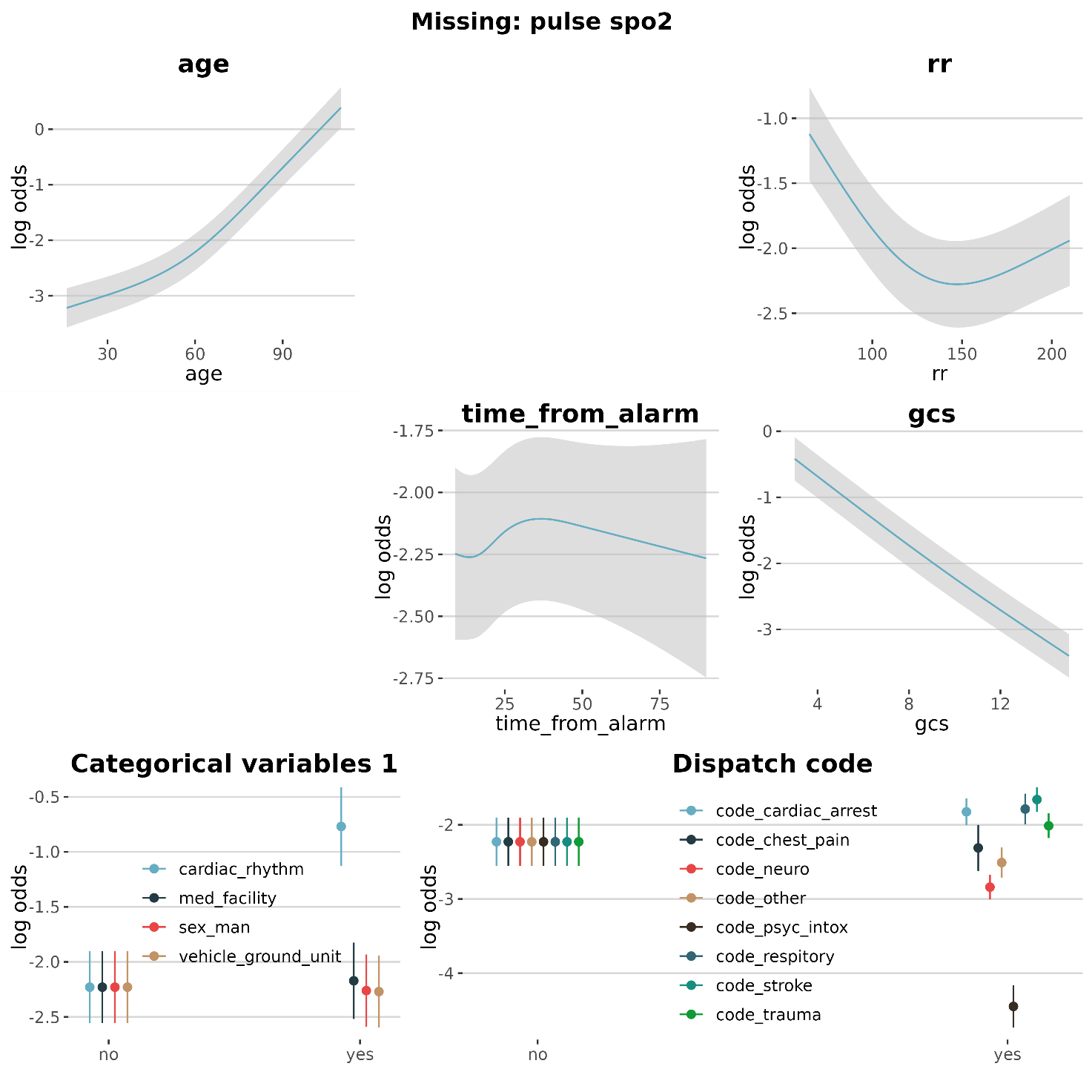

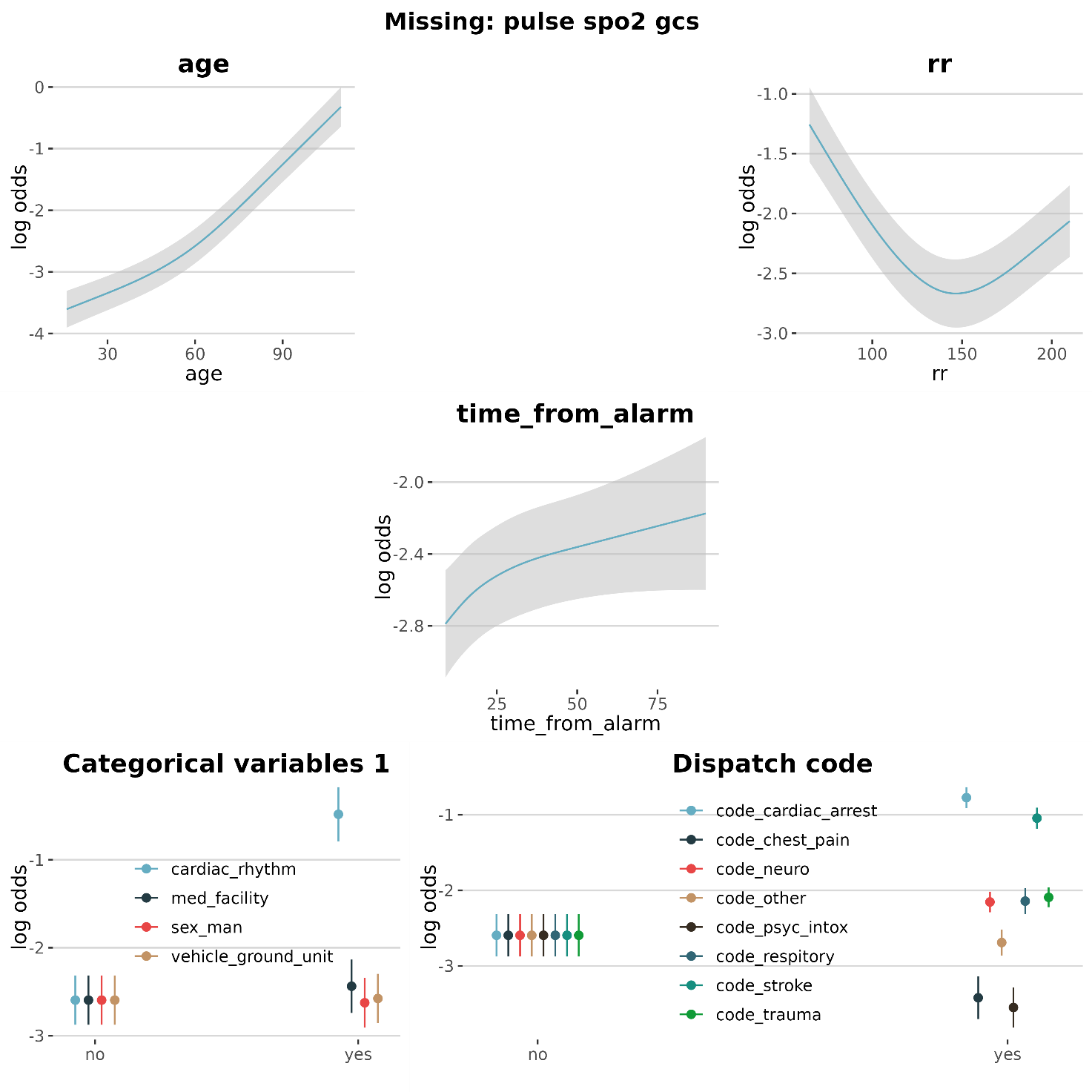

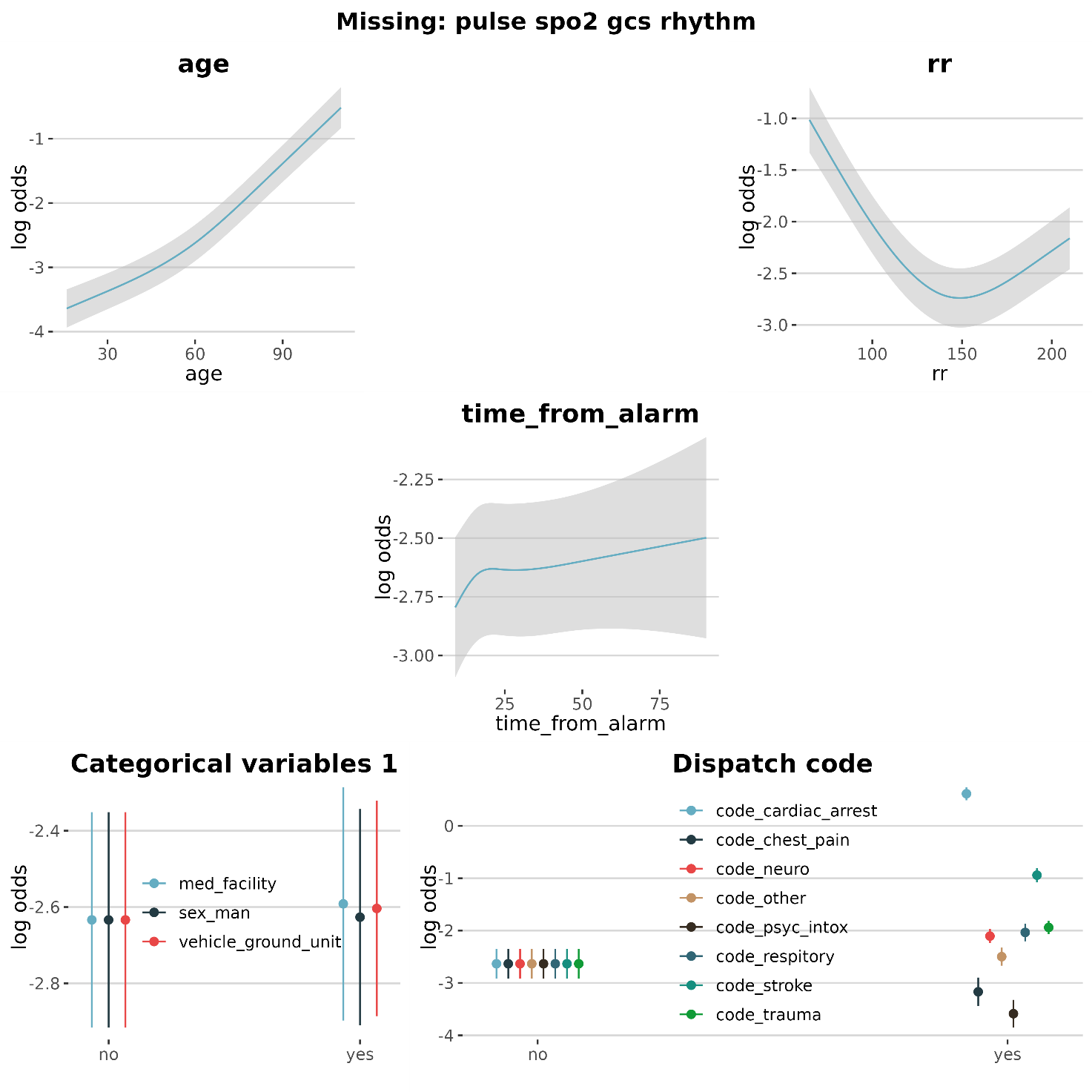

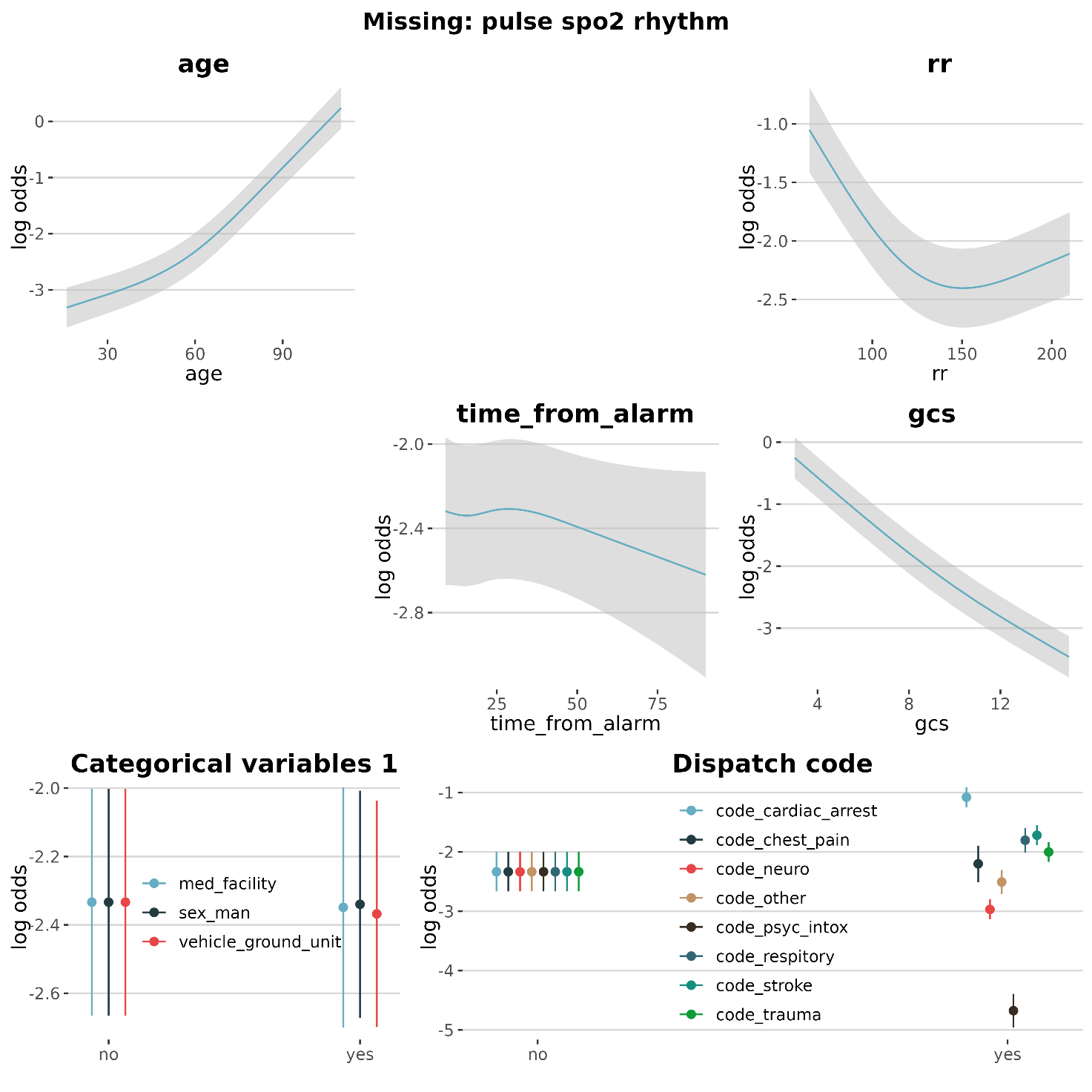

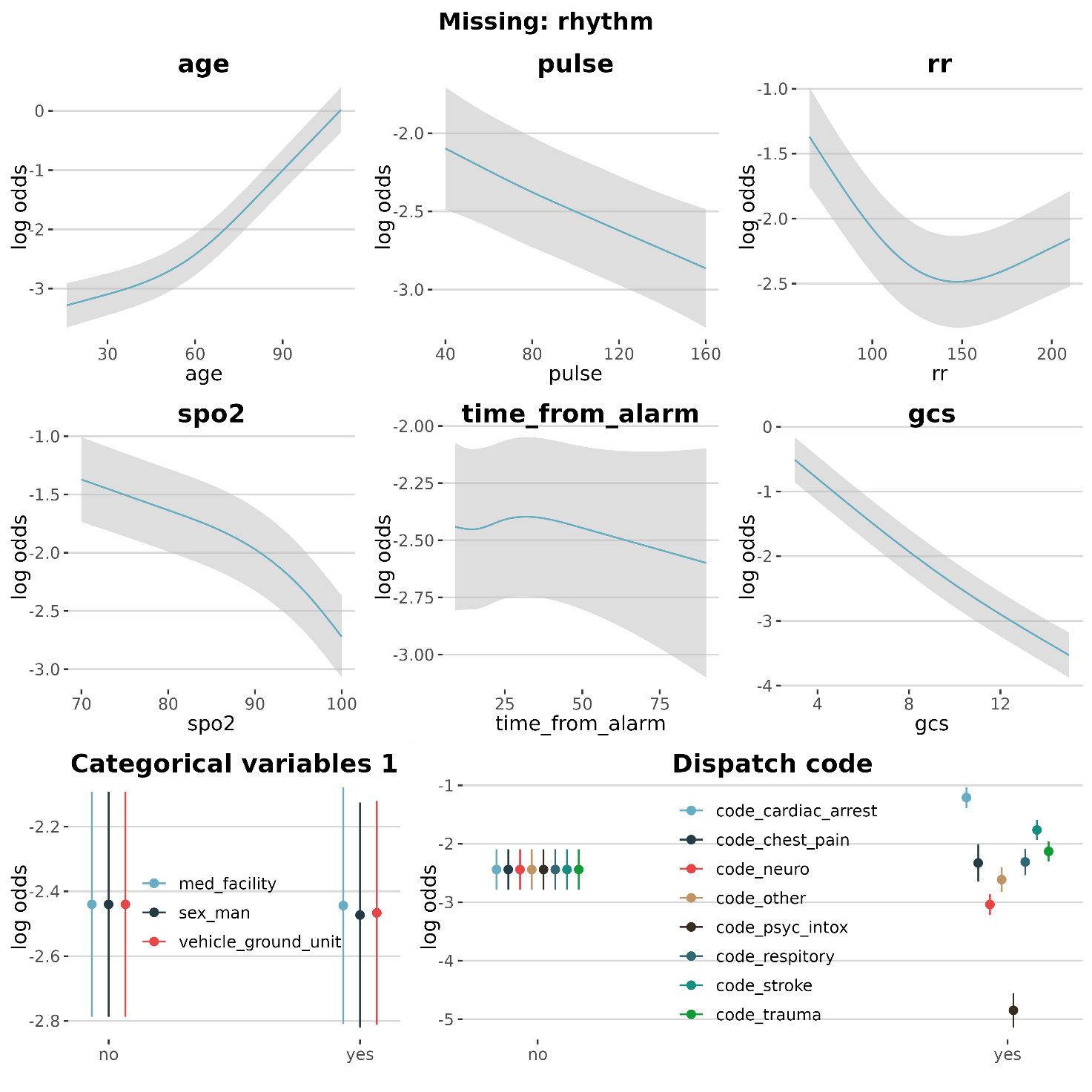

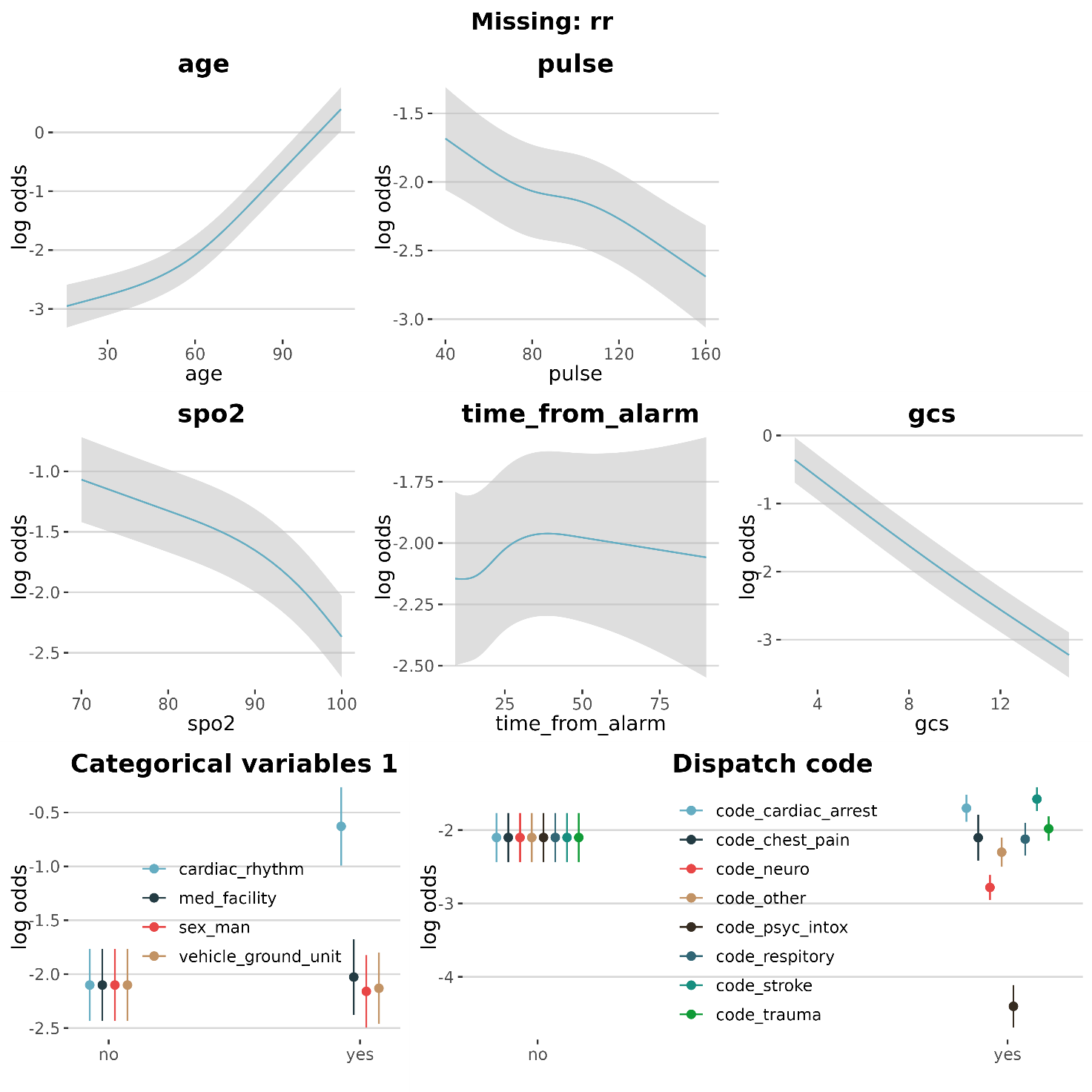

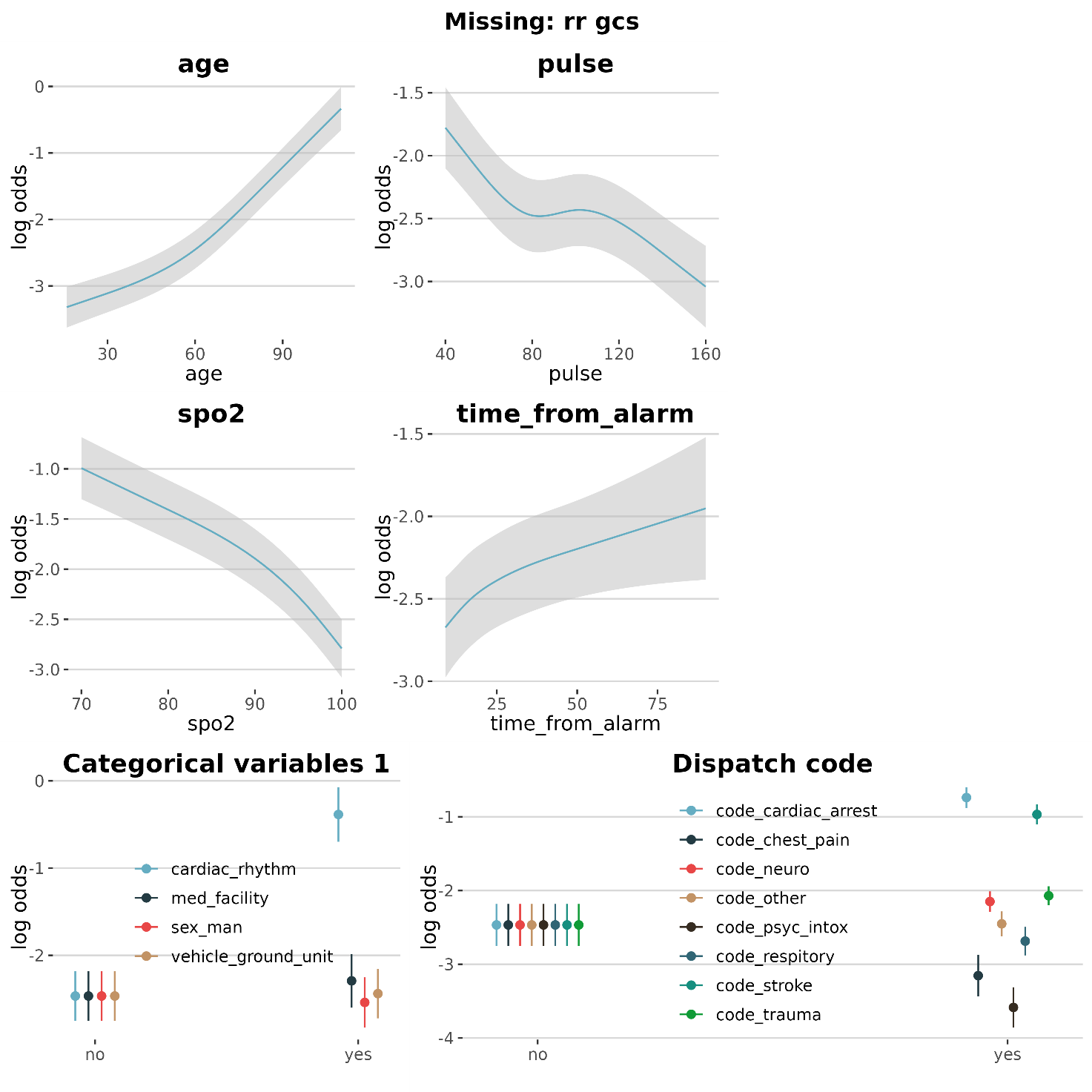

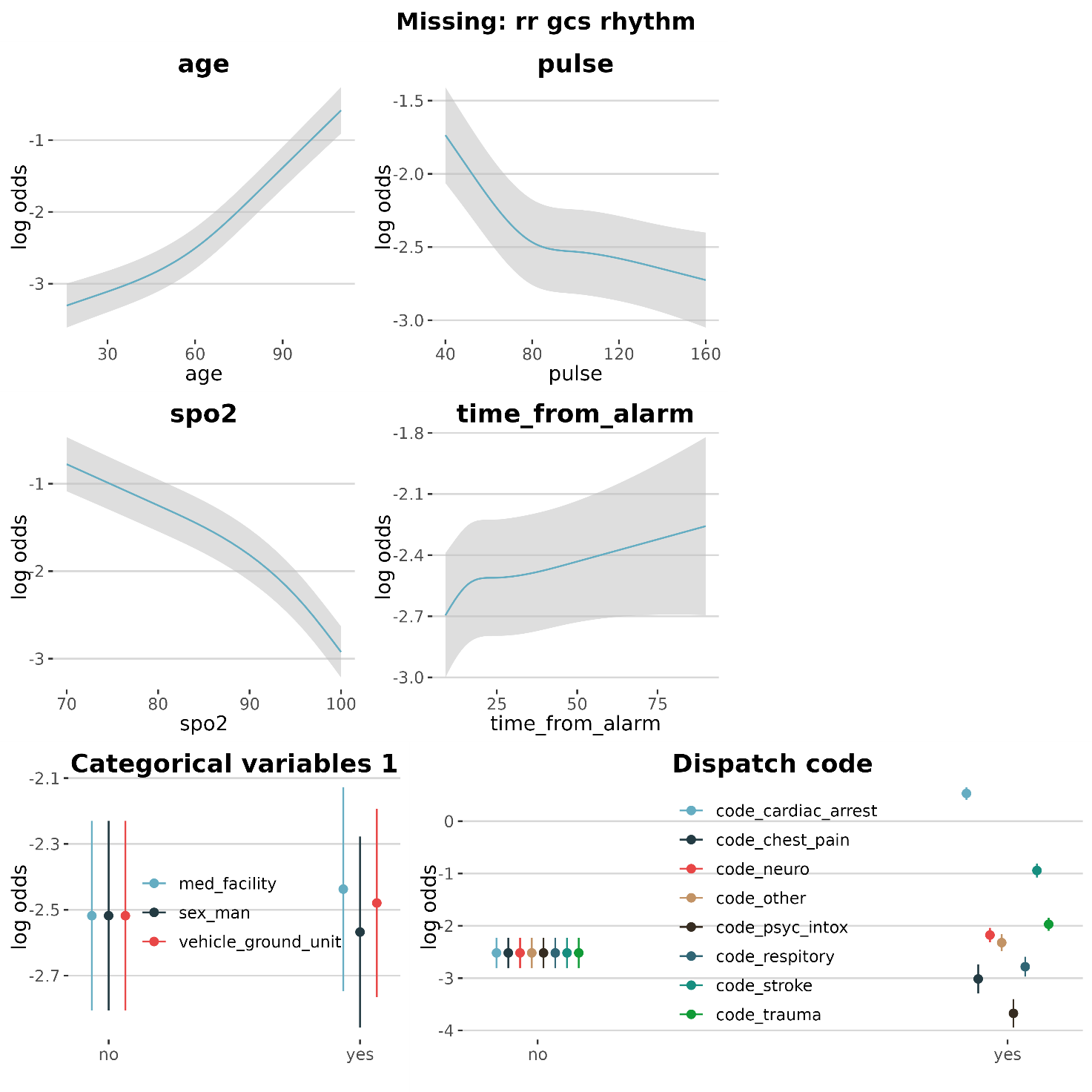

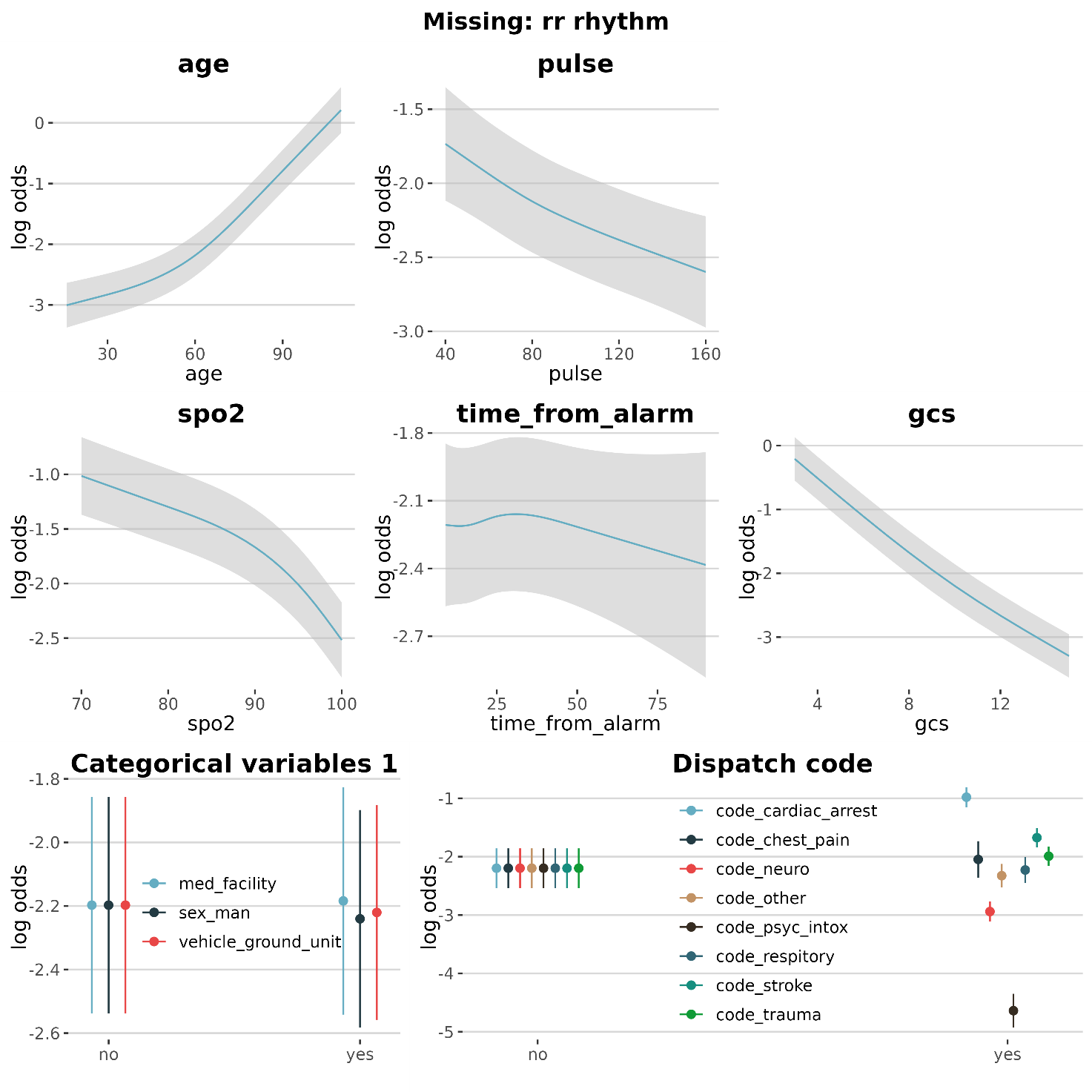

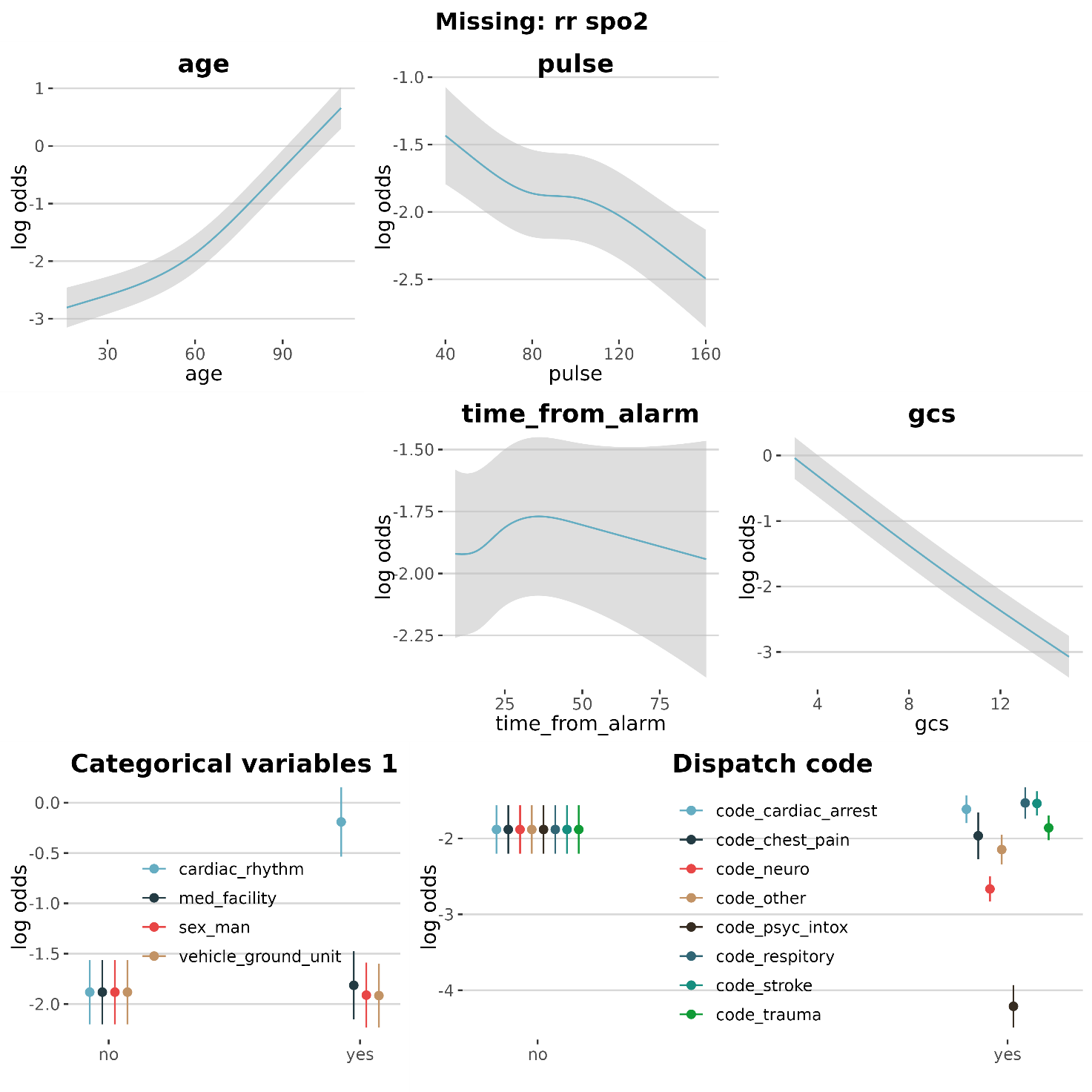

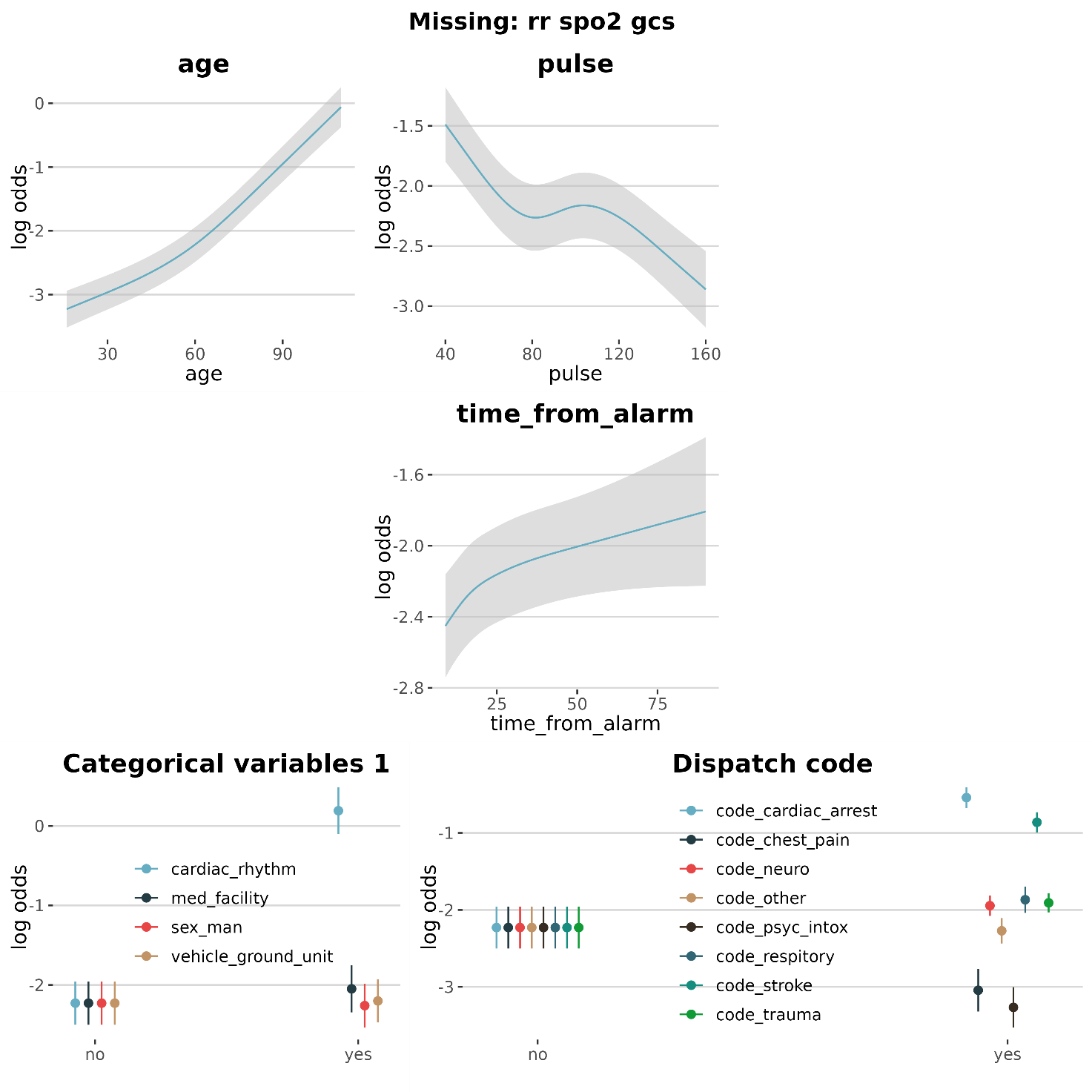

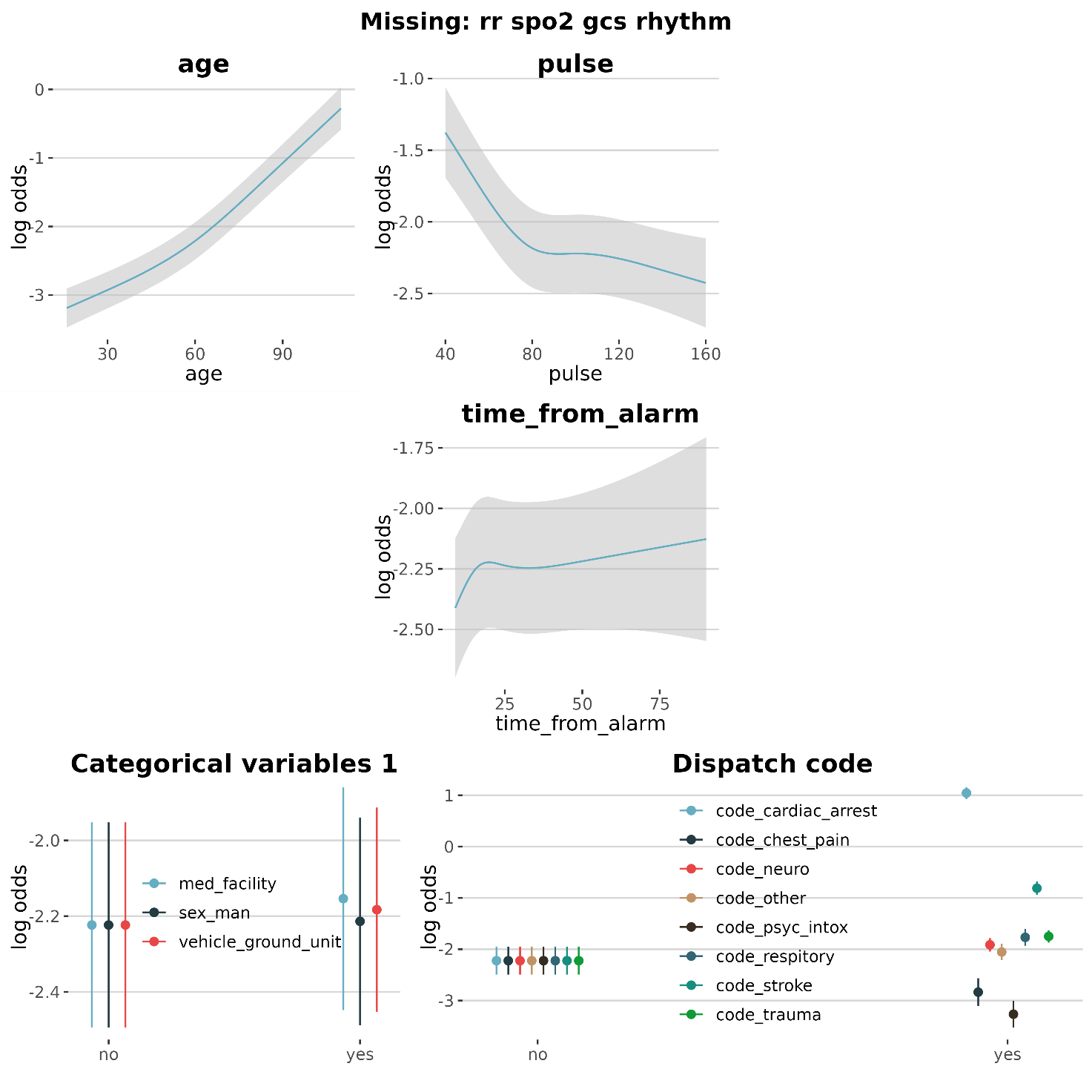

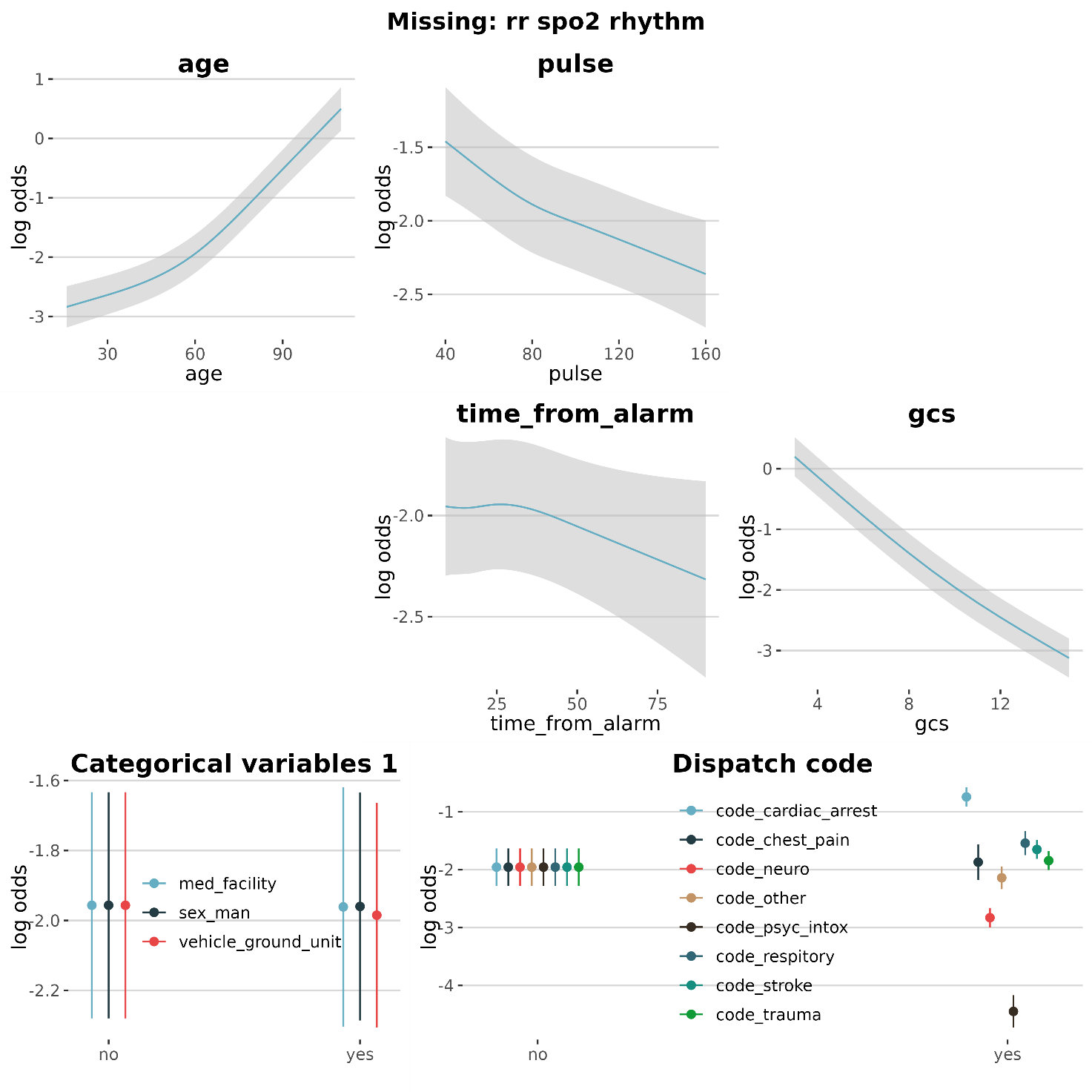

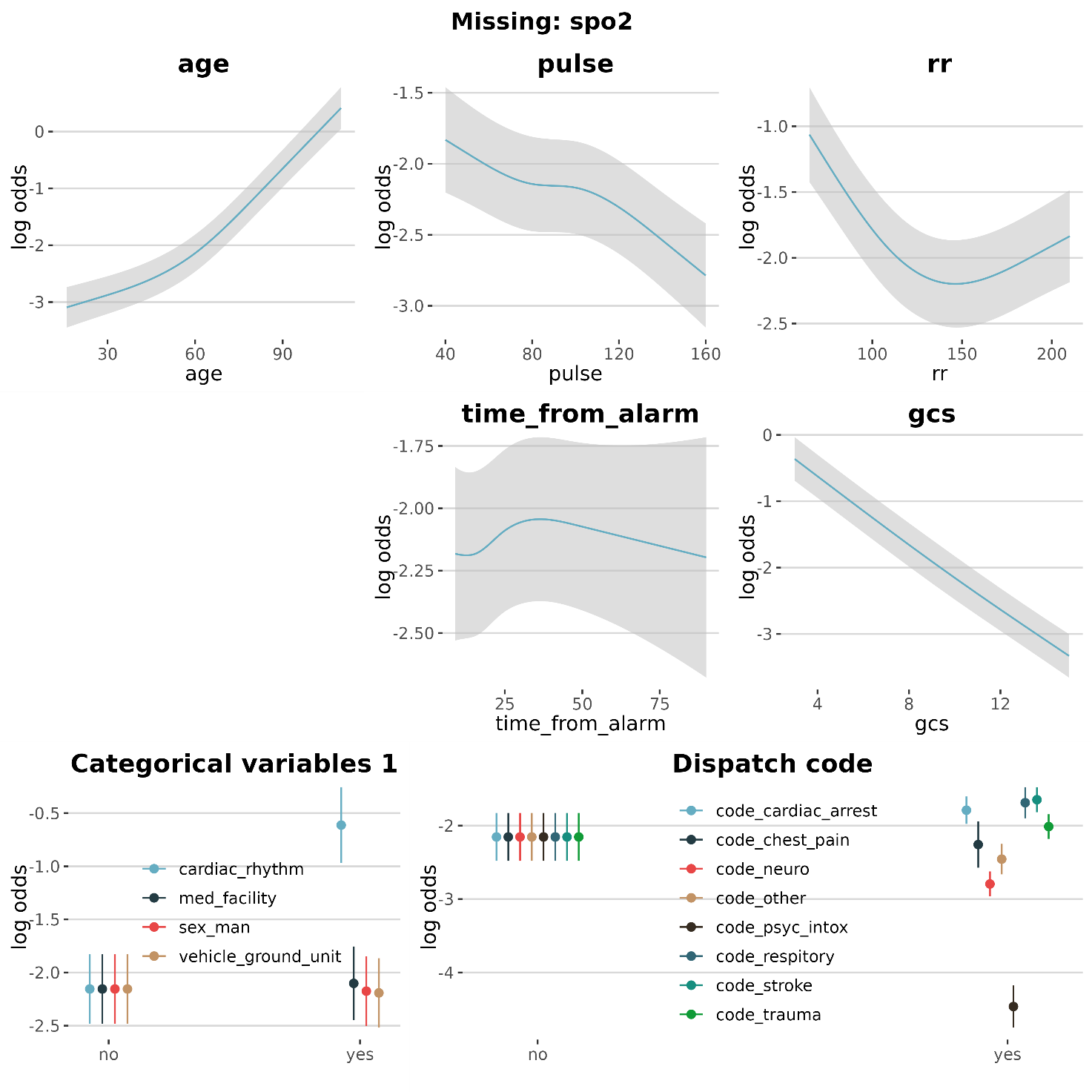

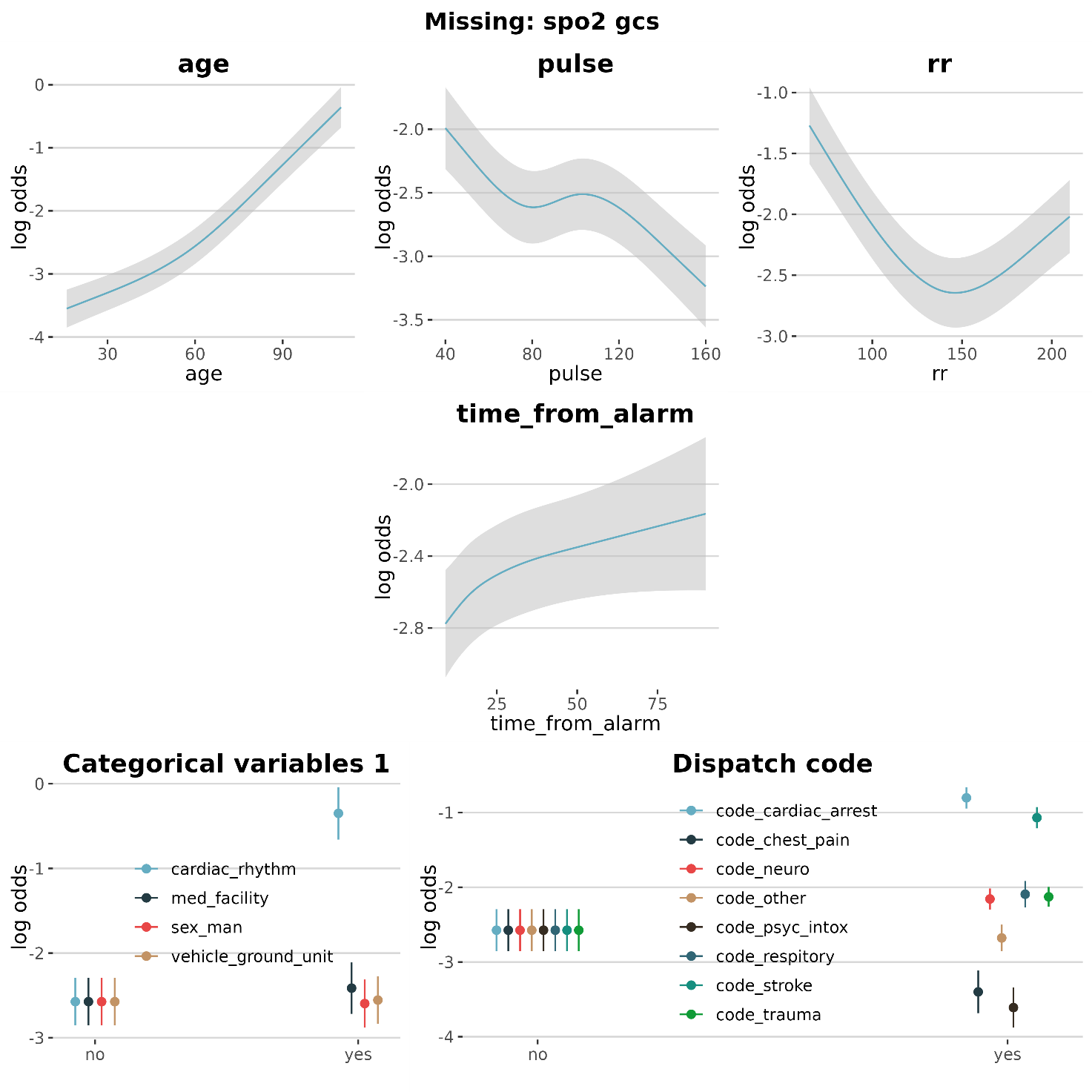

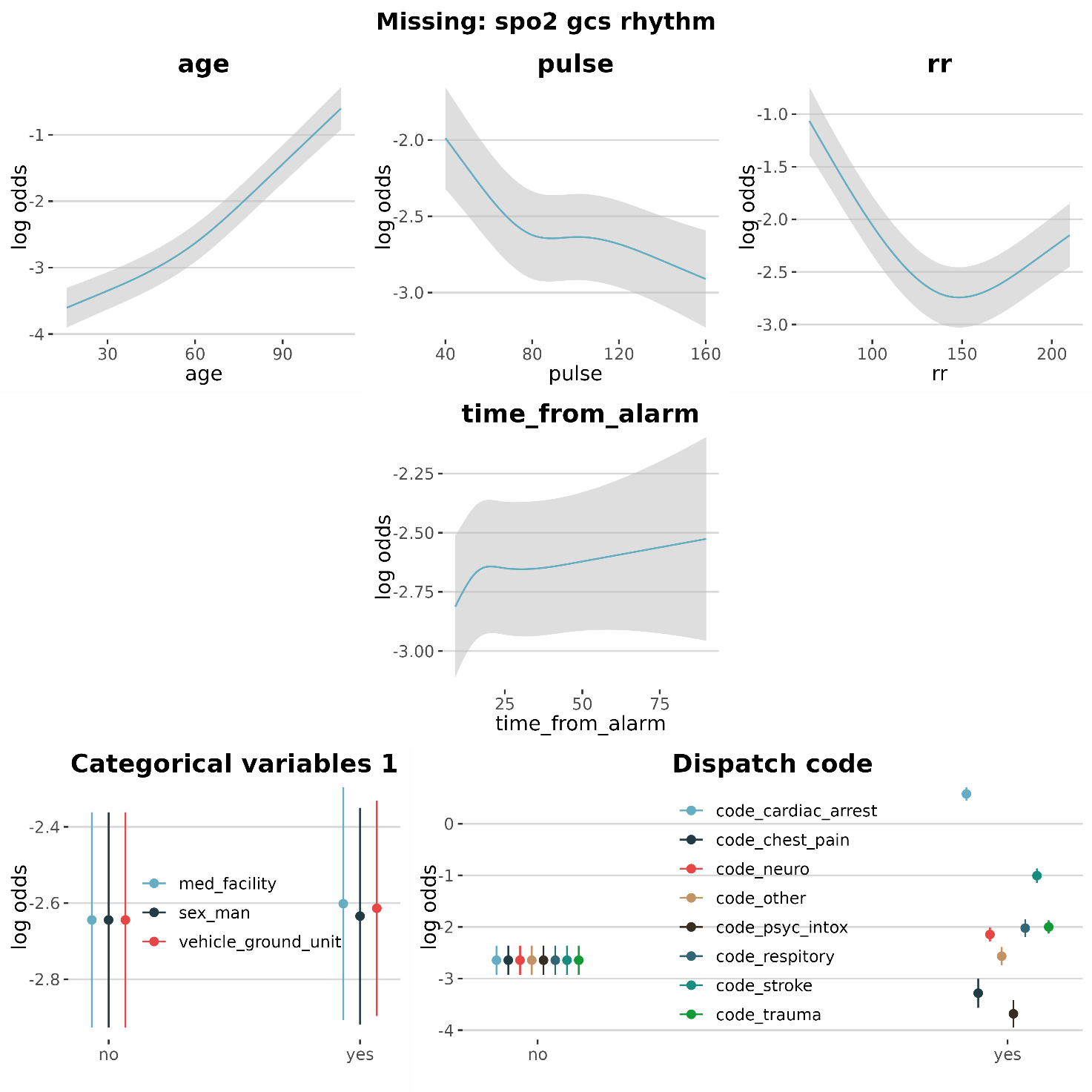

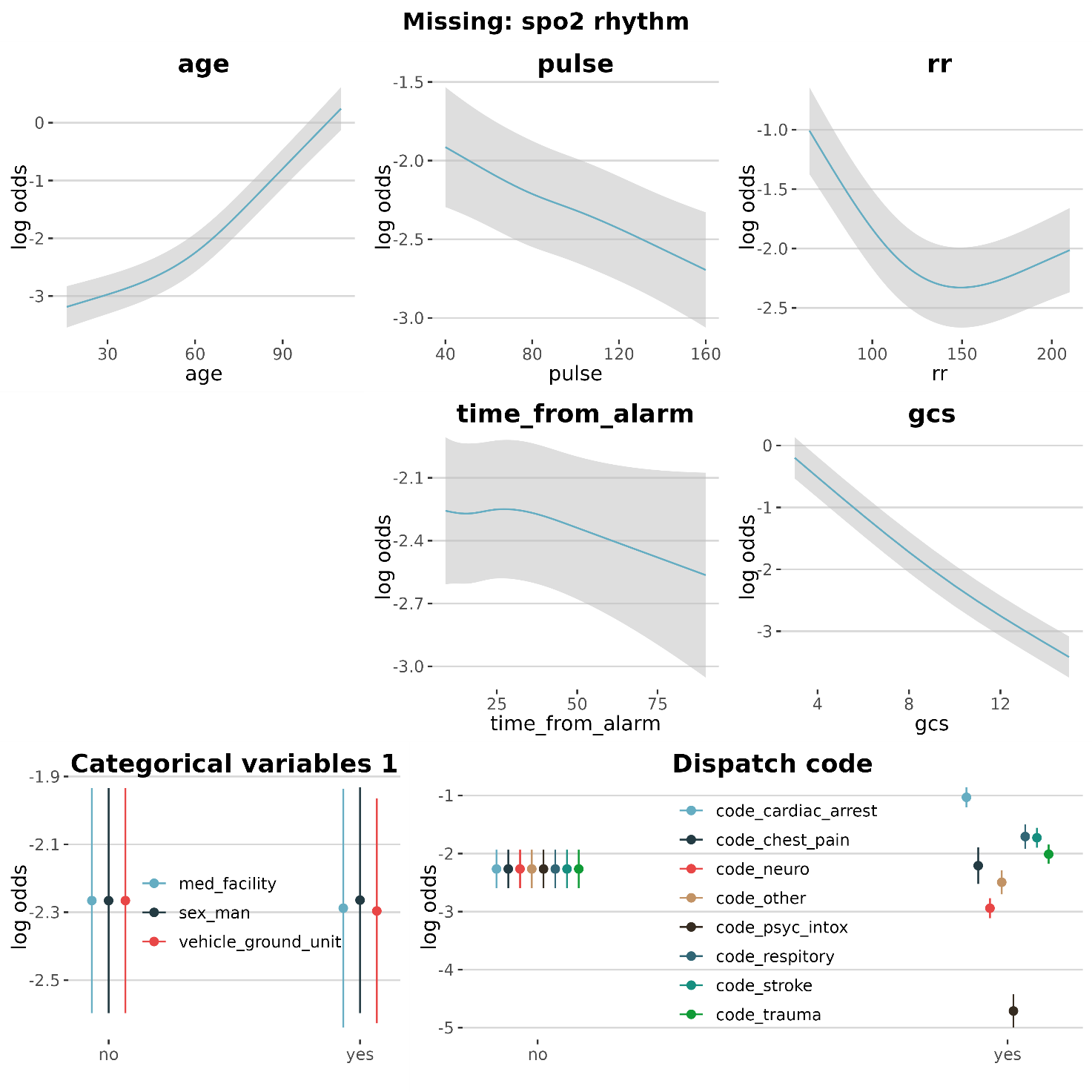

Supplement: Supplementary file 4 — Additional file 4. Estimates for each individual model. [file 13049_2024_1208_MOESM4_ESM.docx]
